# X‐Box Binding Protein 1 Regulates Osteogenesis of Periodontal Ligament Cells During Aging by Modulating P53 Signaling Pathway

**DOI:** 10.1155/sci/2846096

**Published:** 2026-04-10

**Authors:** Youli Zheng, Mengru Zhai, Xijing Bai, Kaiqi Yu, Jia Li, Zheng Zhang

**Affiliations:** ^1^ The School and Hospital of Stomatology, Tianjin Medical University, Tianjin, 300070, China, tijmu.edu.cn; ^2^ Tianjin Stomatological Hospital, School of Medicine, Nankai University, Tianjin, 300000, China, nankai.edu.cn; ^3^ Tianjin Key Laboratory of Oral and Maxillofacial Function Reconstruction and Stomatology Institute of Nankai University, Tianjin, 300041, China

**Keywords:** aging, osteogenesis, periodontal ligament cells, periodontitis, X-box binding protein 1

## Abstract

**Background and Objective:**

Aging impairs the osteogenic differentiation of periodontal ligament stem cells (PDLSCs), but the mechanism is unclear. X‐box binding protein 1 (XBP1), a transcription factor affecting cell senescence, may be involved. This study focuses on the role and mechanism of XBP1 in PDLSCs’ osteogenic differentiation during aging.

**Methods:**

Young PDLSCs (Y‐PDLSCs) and elderly PDLSCs (E‐PDLSCs) were obtained. XBP1 expression was quantified using both real‐time polymerase chain reaction (RT‐PCR) and western blotting (WB). Lentiviral infection was employed to modulate XBP1 expression, with pLVX‐XBP1 utilized for upregulation and shXBP1 for downregulation. The degree of senescence in PDLSCs was determined using β‐galactosidase staining and cell cycle analysis. PDLSCs’ osteogenic differentiation was assessed through WB, alkaline phosphatase (ALP), and alizarin red staining. The Gene Transcription Regulation Database (GTRD) and microarray analysis was used to identify XBP1’s target genes and potential pathways. Additionally, PFTα was applied to inhibit the P53/P21 pathway in PDLSCs.

**Results:**

E‐PDLSCs had higher β‐galactosidase expression, suppressed cell cycle, and decreased osteogenic differentiation compared to Y‐PDLSCs. XBP1 expression in PDLSCs decreased with aging, and upregulation of XBP1 by pLVX‐XBP1 partially reversed this aging process. Osteogenic induction increased XBP1 expression in PDLSCs, and pLVX‐XBP1 facilitated the osteogenic differentiation of these cells. The target genes of XBP1 were enriched in the P53/P21 pathway, which was highly expressed in P9 PDLSCs but showed lower expression following XBP1 overexpression. Moreover, inhibiting the P53/P21 pathway by PFTα could partially reverse the inhibitory effect of shXBP1 on PDLSCs’ osteogenic differentiation.

**Conclusions:**

XBP1 plays a pivotal role in the regulation of PDLSCs’ osteogenic differentiation during the aging process, at least in part, through the modulation of the P53/P21 pathway.

## 1. Introduction

In humans, bone is a highly dynamic tissue that undergoes constant self‐regeneration throughout adulthood to maintain its structural integrity in a process known as bone remodeling [1]. Alveolar bone, one of the most actively remodeled bones in the human body, plays a crucial role in maintaining the health of periodontal tissue and the stability of teeth [2]. However, during periodontitis, the bone remodeling process of alveolar bone is disrupted, manifested as a decrease in osteogenic activity and an increase in osteoclastic activity, which results in the continuous degradation of alveolar bone, eventually causing tooth loosening and even tooth loss [3]. Aging further exacerbates the impairment of bone remodeling, accelerating the destruction of alveolar bone in periodontitis and significantly increasing the complexity of periodontal treatment [2].

Cellular senescence, a key biological phenomenon, is characterized by an irreversible growth halt, triggered by replication capacity exhaustion or stressors like DNA damage, oxidative stress, and inflammation [4]. In the context of periodontitis, senescent cells accumulate over time in the alveolar bone, contributing significantly to the age‐related degradation of this critical structure [5, 6]. Periodontal ligament stem cells (PDLSCs), a type of mesenchymal stem cells isolated from the periodontal ligament, play a crucial role in maintaining periodontal homeostasis during the healing process following dental injuries [7]. However, research has shown that the viability and osteogenic differentiation potential of PDLSCs tend to decline with advancing age [8], which may significantly contribute to the development and progression of age‐related periodontal diseases.

The dysregulation of transcription factors pivotal in bone development is a paramount factor contributing to the aging process of bone‐resident mesenchymal stem cells [1]. Among these transcription factors, X‐box binding protein 1 (XBP1) emerges as a critical regulator of longevity in a cell‐nonautonomous manner [9]. It exerts its longevity‐promoting effects through both the canonical unfolded protein response (UPR) endoplasmic reticulum (ER) and non‐canonical pathways in distinct tissues [10]. Recent investigations have underscored the crucial role of XBP1 in the aging dynamics of various cells and organs [10–14]. However, its impact on PDLSCs senescence remains unclear. In the present study, we aimed to unravel the alterations in the expression profile of XBP1 during the aging progression of PDLSCs and elucidate the effects of XBP1 on the aging process and osteogenic differentiation capacity of these cells.

## 2. Materials and Methods

### 2.1. Isolation of PDLSCs

Young PDLSCs (Y‐PDLSCs) were isolated from donors aged 15–25, and elderly PDLSCs (E‐PDLSCs) from 55 to 75. With the approval of the Ethics Committee of Tianjin Stomatological Hospital (PH2023‐J‐012), periodontal ligament tissues were collected and digested with 0.2% type I collagenase (Gibco, USA) at 37°C for 2 h. The resulting suspensions were cultured in α‐MEM medium (Gibco, USA) supplemented with 10% fetal bovine serum (FBS; Gibco, USA), 100 μg/mL streptomycin (Beyotime, China), and 100 U/mL penicillin (Beyotime, China) at 37°C in a 5% CO_2_ environment. Third passage PDLSCs (P3), as well as P6 and P9 cells following passaging, were utilized in subsequent experiments. The characterization of the PDLSCs (P3) was verified by flow cytometry.

### 2.2. Cell Transfection

The lentiviral vector pLVX‐IRES‐Puro (GenePharma, Shanghai, China) and oligonucleotide sequences mimicking XBP1 were employed to construct the pLVX‐XBP1 plasmid. Additionally, the negative sham oligonucleotides for the XBP1 (pLVX‐NC) were also synthesized. Plasmids, including pLV3‐U6‐XBP1‐shRNA1, pLV3‐U6‐XBP1‐shRNA2, pLV3‐U6‐XBP1‐shRNA3, and matched control mocks (shRNA NC) were from Miaolingbio (Wuhan, China). Lentiviral constructs were made by Tsingke (Beijing, China). Lentivirus supernatants were applied to infect PDLSCs and puromycin was utilized to select target cells after 72 h of infection.

### 2.3. XBP1 Target Genes Screening and Enrichment Analysis

GSE159507 (mRNA‐seq) from the GEO database (http://www.ncbi.nlm.nih.gov/geo/) was employed to screen osteogenic genes and contained six PDLSCs samples. GEO2R online tool was utilized to identify differentially expressed mRNAs (DE‐mRNAs). We set |log_2_ fold change (FC)| > log_2_ 1.5 and *p*‐value < 0.05 as the thresholds for identifying DE‐mRNAs. XBP1 target genes in PDLSCs were identified by conducting a combined analysis of upregulated DE‐mRNAs in GSE159507 and XBP1 targets from the Gene Transcription Regulation Database (GTRD; http://gtrd.biouml.org). The webgestalt database (http://www.webgestalt.org/) was used for Gene Ontology (GO) and Kyoto Encyclopedia of Genes and Genomes (KEGG) pathway enrichment analyses, respectively.

### 2.4. β‐Galactosidase Staining Assay

Begin by digesting the PDLSCs and inoculating them into a six‐well plate at a density of approximately 1 × 10^4^ cells/mL. Cultivate until cells reach 50%–60% confluence, then remove the medium and rinse with PBS. Fix the cells at room temperature for 15 min using the fixative from the β‐galactosidase staining kit (Solarbio, China). Discard the fixative and rinse three times with PBS. Stain the cells with freshly prepared β‐galactosidase solution and incubate at 37°C for 12 h. Finally, observe under a regular optical microscope and count the positive cells.

### 2.5. Quantitative Real‐Time Polymerase Chain Reaction (RT‐PCR)

Total RNA was extracted from PDLSCs using Trizol (Invitrogen, USA), and reverse‐transcribed into cDNA with the PrimeScript RT reagent Kit (Takara, Japan). RT‐qPCR was performed using TB Green Premix Ex Taq (Takara, Japan) on the Roche Light Cycler 480 (Roche Diagnostics, Germany). GAPDH was used as an internal reference, and mRNA expression was calculated using the 2^−ΔΔCt^ method. Primer sequences are shown in Table S1.

### 2.6. Analysis of Cell Cycle

After replacing the culture medium with serum‐free basal medium to synchronize the cells, they were digested and collected. The cells were then washed twice with pre‐cooled PBS and fixed overnight at 4°C with 70% alcohol. After washing, cell cycle staining solution (Bioss, Beijing, China) was added, and the cells were stained in the dark at 37°C for 20 min. Finally, flow cytometry was used for cell cycle analysis.

### 2.7. Western Blotting (WB)

Proteins were extracted from PDLSCs using a protein lysate (Beyotime, China), and centrifuged at 12,000 rpm for 10 min. Samples were then separated on 10% SDS–PAGE gels, transferred to PVDF membranes (Millipore, USA), and blocked with 5% non‐fat milk. Primary antibodies were incubated overnight at 4°C, followed by secondary antibodies for 1 h at room temperature. ECL solution was used for visualization. The quantitative analysis was performed using ImageJ software. Primary antibodies included osteocalcin (OCN, Abcam, UK), Runt‐related transcription factor‐2 (Runx2, Abcam, UK), XBP1 (proteintech, USA), P21 (proteintech, USA), P53 (proteintech, USA), and GAPDH (proteintech, USA).

### 2.8. Alkaline Phosphatase (ALP) Staining

After 14 days of osteogenic induction, the PDLSCs were fixed in 70% ethanol for 30 min and washed twice with distilled water. Following this, the cells were stained for ALP activity at room temperature using an ALP Staining Kit (KeyGEN, China), according to the manufacturer’s instructions. The stained cells were then observed and photographed under a microscope. To quantify ALP activity, the cells were lysed with 1% Triton X‐100, and the absorbance of the samples was measured at 520 nm using a spectrophotometer.

### 2.9. Alizarin Red Staining

Following a 14‐day incubation period in an osteogenic medium, the PDLSCs were fixed with 4% paraformaldehyde for 30 min and subsequently rinsed three times with distilled water. The fixed PDLSCs were then stained with a 2% alizarin red solution (Leagene, China) for 30 min and examined using both photographic and microscopic techniques. Additionally, 10% acetic acid was employed to solubilize the calcium‐bound alizarin red, and the absorbance of the released dye was quantified at 405 nm utilizing spectrophotometric analysis.

### 2.10. Chromatin Immunoprecipitation (ChIP)

ChIP analysis was performed to detect XBP1 binding to the *P53* promoter. PDLSCs were cross‐linked with 1% formaldehyde, quenched with 0.125 mol/L glycine, washed with PBS, resuspended in lysis buffer, and sonicated to fragment DNA. Lysates were centrifuged (20,000 g, 10 min, 4°C), and supernatants were immunoprecipitated with antibody against XBP1 (proteintech, USA). The DNA–protein complex was precipitated and quantified using qRT‐PCR using specific primers (Table S2). Immunoglobulin G acted as a negative control.

### 2.11. Luciferase Assay

Luciferase assay was employed to ascertain the activity of the *P53* promoter. The pGL3 luciferase reporter plasmid, containing the *P53* promoter region (mutant and wild types), was co‐transfected with the XBP1 overexpression plasmid into PDLSCs using Lipofectamine 2000. Following a 24‐h post‐transfection period, the cells were harvested, and the luciferase activity was quantified using a dual‐luciferase reporter assay system (YEASEN, China).

### 2.12. Statistical Analysis

All data were expressed as the means ± standard deviation (SD). Unpaired *t*‐tests were used for two‐group comparisons, and one‐way ANOVA with Bonferroni corrections for multiple groups. *p*‐Value < 0.05 was considered as statistically significant. All statistical analyses were performed using SPSS 23.0 (IBM Corp., USA).

## 3. Results

### 3.1. PDLSCs Derived From Elderly Individuals Exhibit Aging Characteristics

Both Y‐PDLSCs and E‐PDLSCs displayed negative expression of hematopoietic markers CD34 and CD45, but exhibited strong positive expression of mesenchymal‐associated markers CD90 and CD146 (Figure 1A,B). Following morphological assessment of primary cultured PDLSCs (Figure 1C) and second‐passage (P2) cells (Figure 1D), Oil Red O (Figure 1E) and alizarin red (Figure 1F) staining were performed to evaluate adipogenic and osteogenic differentiation, respectively.

Figure 1Primary cultured human PDLSCs and cell identification. Flow cytometry analysis of surface markers for (A) young PDLSCs and (B) elderly PDLSCs, respectively. (C) Morphology of primary cultured PDLSCs. (D) Morphology of second‐passage (P2) cultured PDLSCs. (E) Adipogenic differentiation of PDLSCs. (F) Osteogenic differentiation of PDLSCs.

**Figure:**
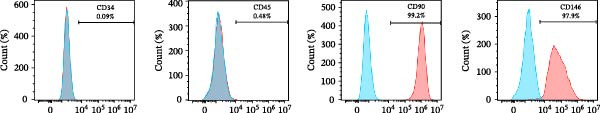
(A)

**Figure:**
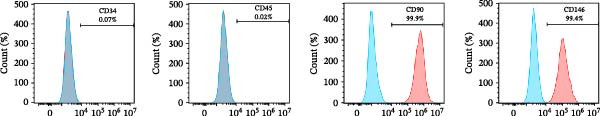
(B)

**Figure:**
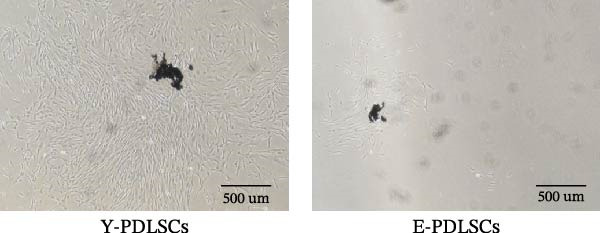
(C)

**Figure:**
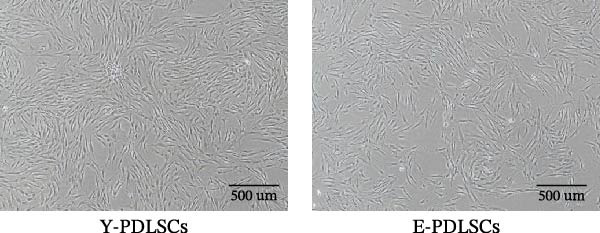
(D)

**Figure:**
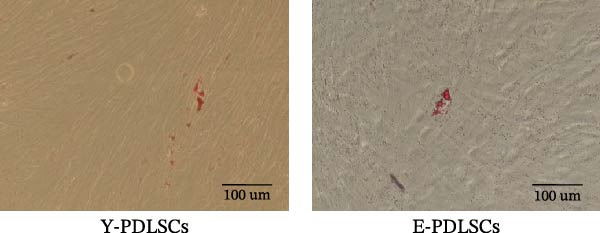
(E)

**Figure:**
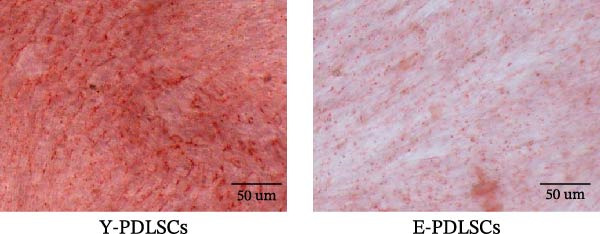
(F)

The activity of the aging marker β‐galactosidase is significantly higher in E‐PDLSCs compared to Y‐PDLSCs (Figure 2A,B). Furthermore, when compared with Y‐PDLSCs, E‐PDLSCs demonstrate an increase in the G1 phase and a decrease in the S phase (Figure 2C,D). These findings suggest that aging impairs the cell cycle progression of PDLSCs.

Figure 2Aging impairs the osteogenic differentiation potential of PDLSCs. (A, B) PDLSCs were stained with β‐galactosidase. (C, D) The cell cycle of PDLSCs was detected by flow cytometry. (E–G) Protein expression levels of RUNX2 and OCN detected by western blotting. (H, I) PDLSCs were stained with alkaline phosphatase (ALP). (J, K) PDLSCs were stained with alizarin red. All experiments were performed in triplicate, and the bars represent the mean ± SD,  ^∗^ *p* < 0.05,  ^∗∗^ *p* < 0.01.

**Figure:**
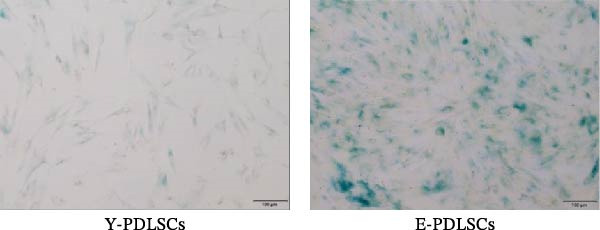
(A)

**Figure:**
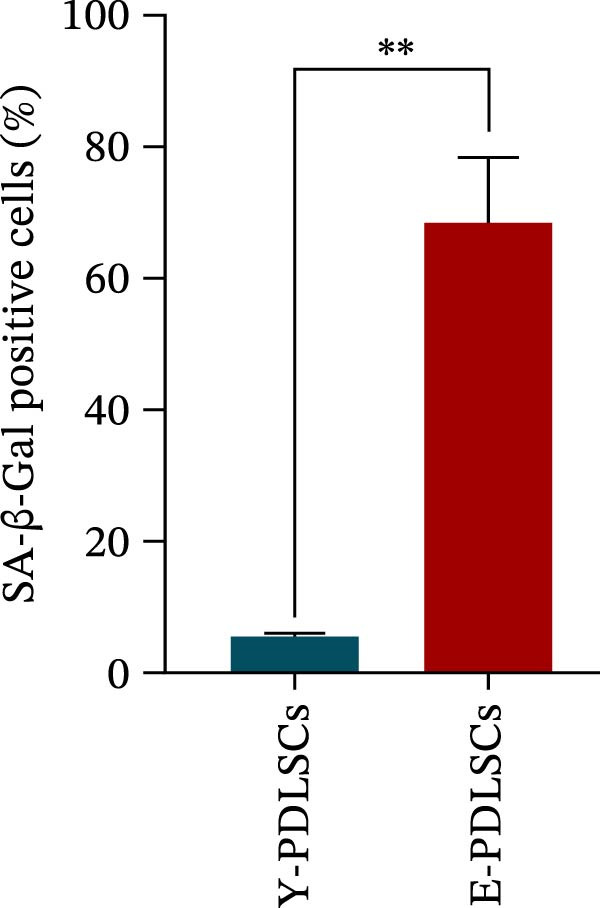
(B)

**Figure:**
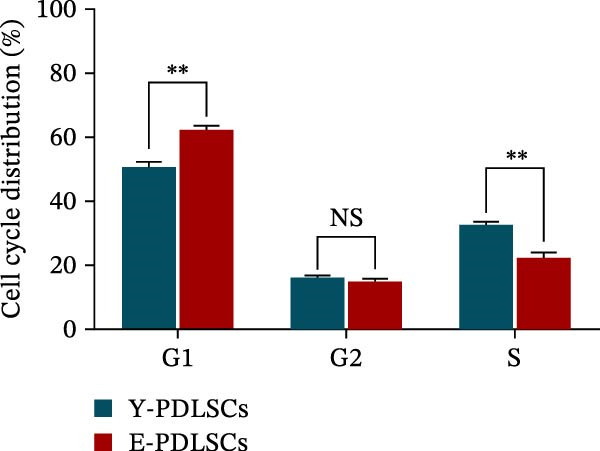
(C)

**Figure:**
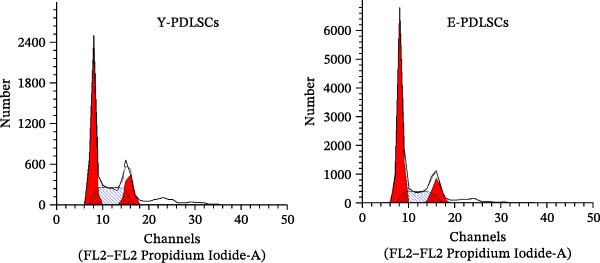
(D)

**Figure:**
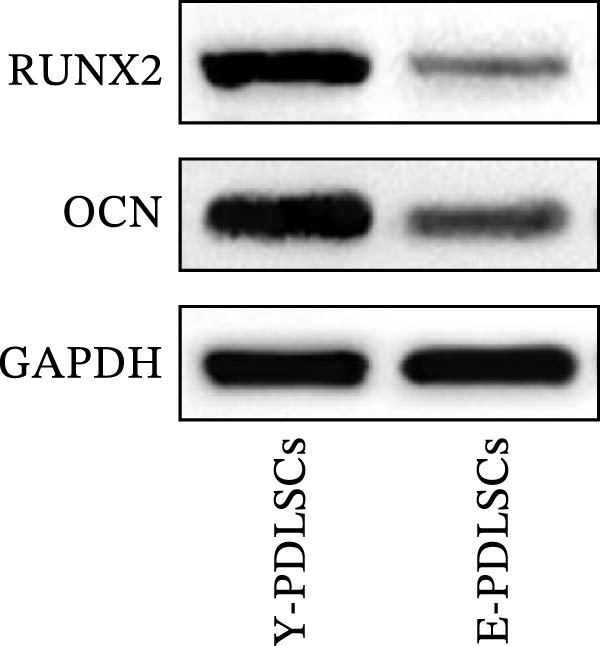
(E)

**Figure:**
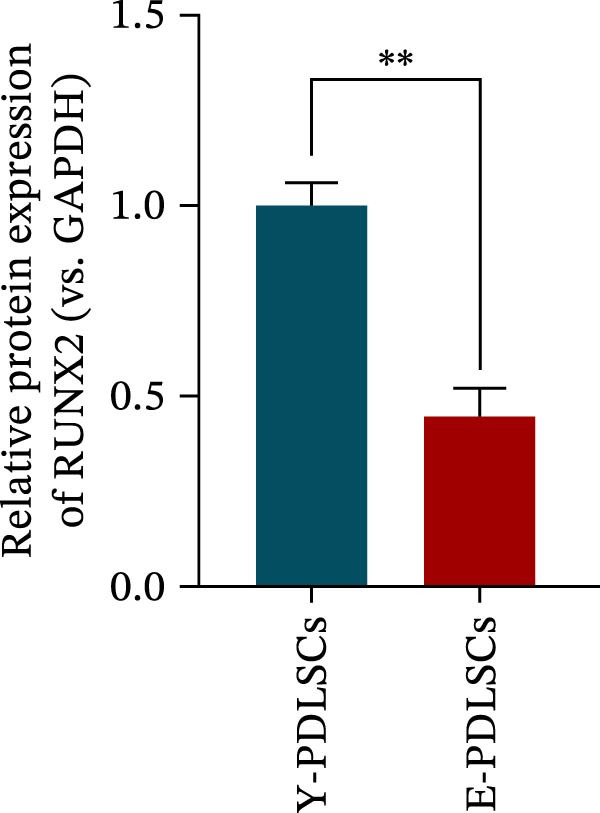
(F)

**Figure:**
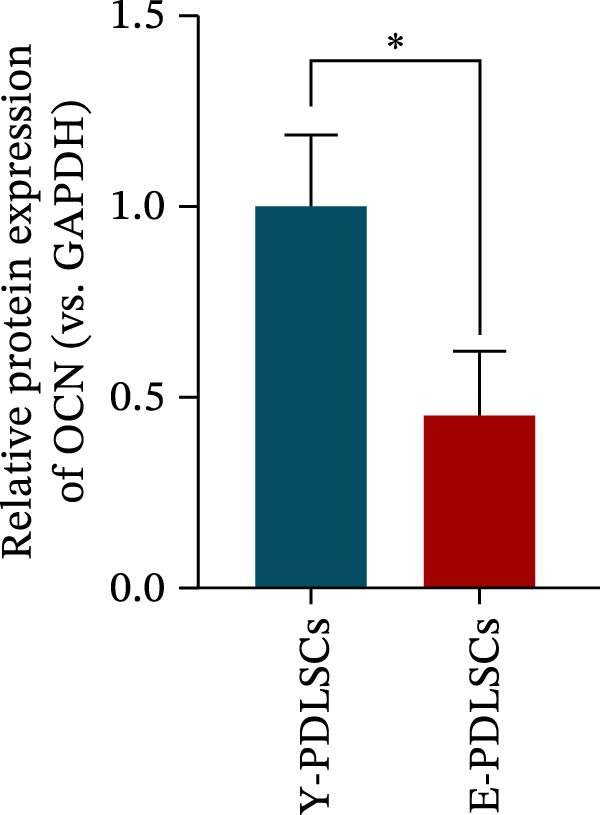
(G)

**Figure:**
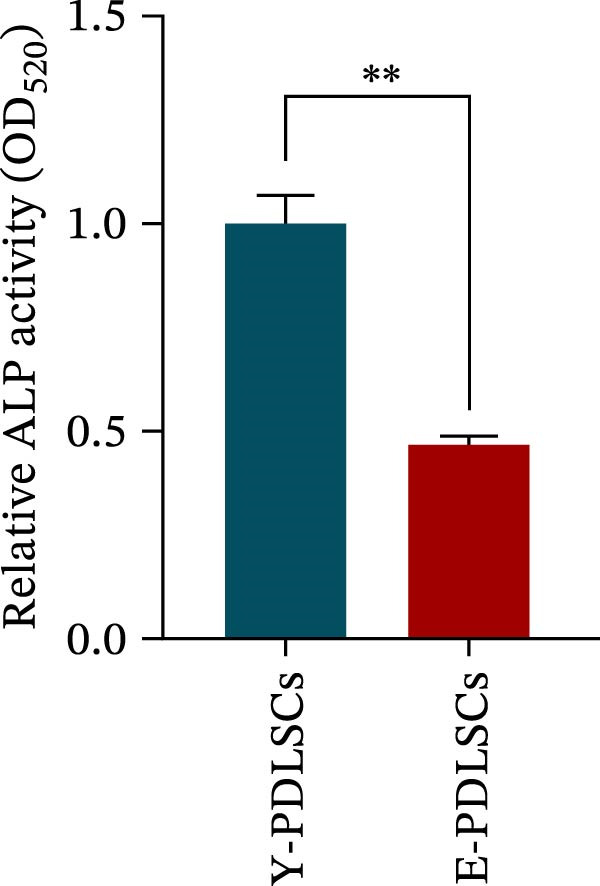
(H)

**Figure:**
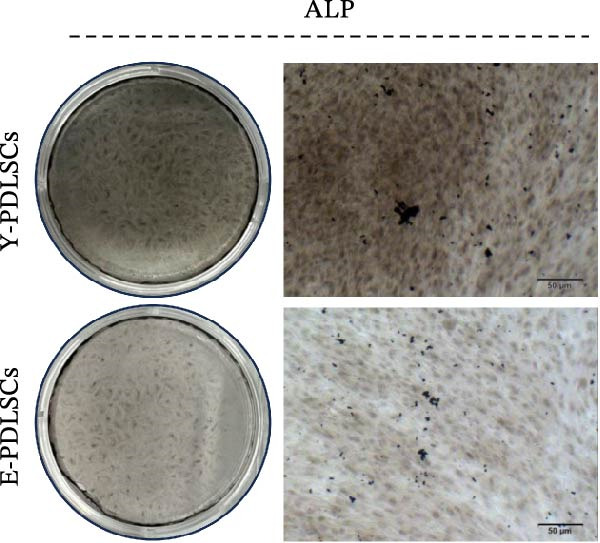
(I)

**Figure:**
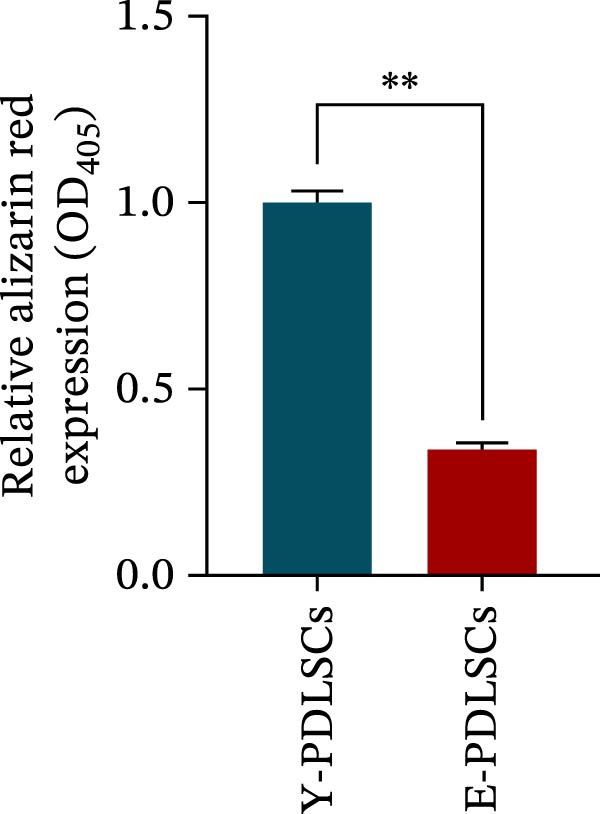
(J)

**Figure:**
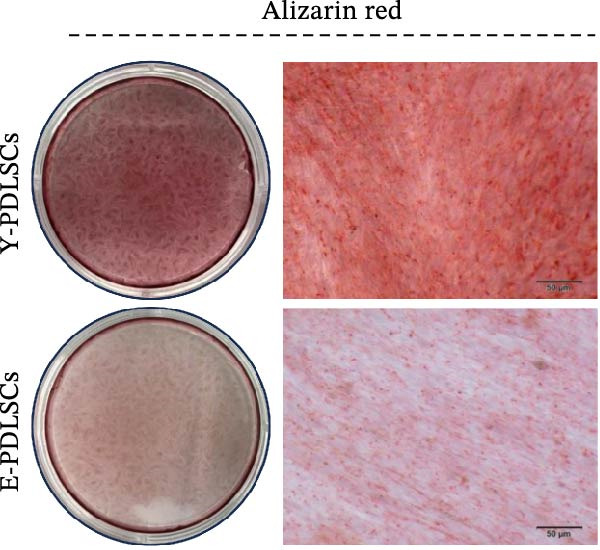
(K)

### 3.2. Aging Impairs the Osteogenic Differentiation Potential of PDLSCs

To explore the impact of aging on the osteogenic differentiation capacity of PDLSCs, we induced osteogenesis in both Y‐PDLSCs and E‐PDLSCs cells. After undergoing osteogenic differentiation, the E‐PDLSCs exhibited downregulated expression of Runx2 and OCN (Figure 2E–G), decreased ALP activity (Figure 2H,I), and reduced mineralization (Figure 2J,K), indicating impaired osteogenic differentiation potential.

### 3.3. XBP1 is Involved in the Aging Process of PDLSCs

The expression level of XBP1 in aging PDLSCs was further investigated, and it was found that XBP1 showed a decreasing trend in E‐PDLSCs (Figure 3A–C). During the replicative aging process of PDLSCs, the level of XBP1 was also monitored. It was observed that the expression level of XBP1 gradually declined with successive passages of PDLSCs (Figure 3D–F). pLVX–XBP1 plasmid was synthesized to overexpress XBP1 in Y‐PDLSCs (Figure 3G). It was discovered that the overexpression of XBP1 significantly reduced β‐galactosidase activity in P9 PDLSCs (Figure 3H,I). Concurrently, the overexpression of XBP1 facilitated the cell cycle progression of P9 PDLSCs, primarily manifested as a decrease in the G1 phase and an increase in the S phase (Figure 3J,K).

Figure 3XBP1 is involved in the aging process of PDLSCs. (A–C) Western blotiing (A, B) and RT‐PCR (C) analysis of XBP1 in young and elderly PDLSCs. Western blotiing (D, E) and RT‐PCR (F) analysis of XBP1 in PDLSCs after different passage. (G) RT‐PCR analysis of XBP1 in Y‐PDLSCs (P3) infected with lentivirus. Following lentiviral infection of Y‐PDLSCs (P3) and subsequent expansion to the P9 generation, cells were subjected to β‐galactosidase staining (H, I), and cell cycle distribution was then assessed by flow cytometry (J, K).  ^∗^ *p* < 0.05,  ^∗∗^ *p* < 0.01.

**Figure:**
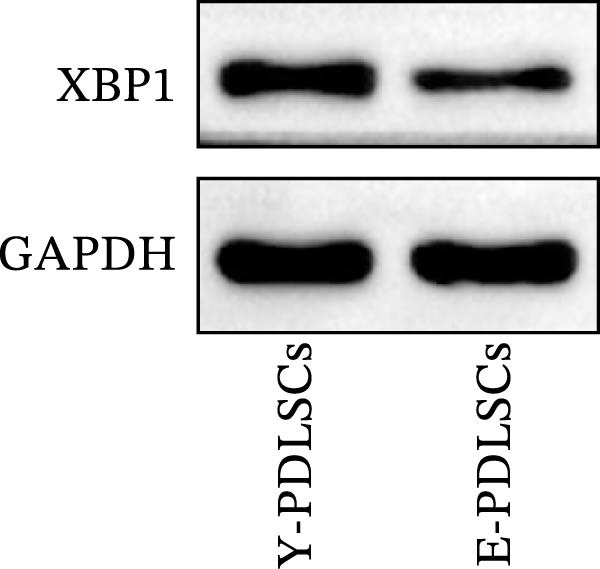
(A)

**Figure:**
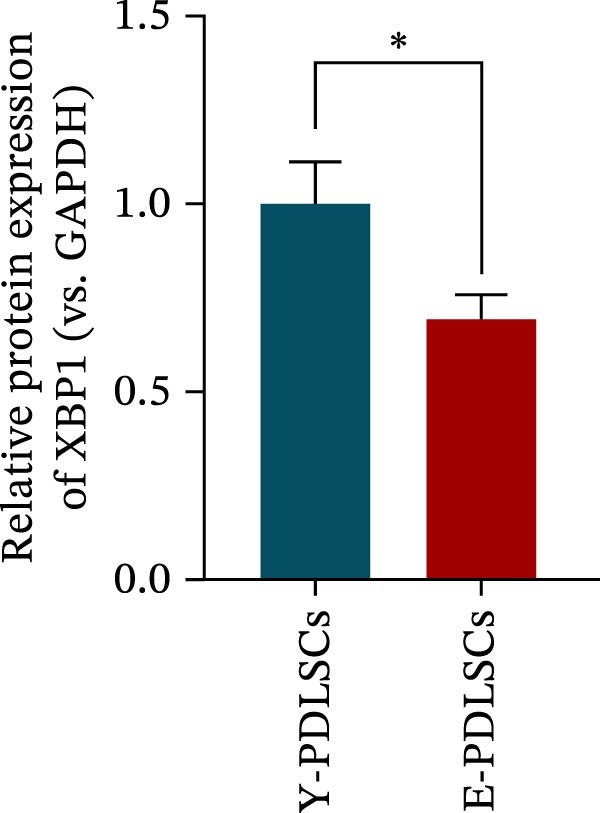
(B)

**Figure:**
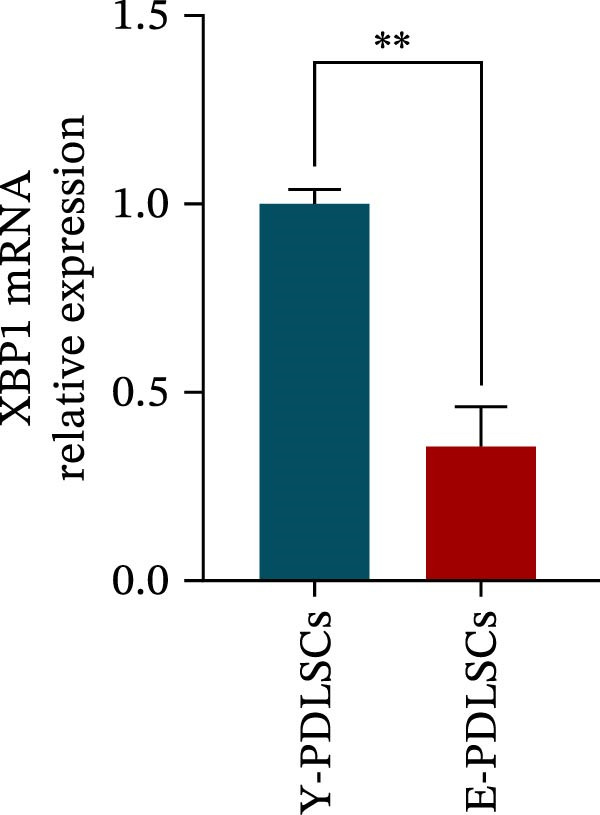
(C)

**Figure:**
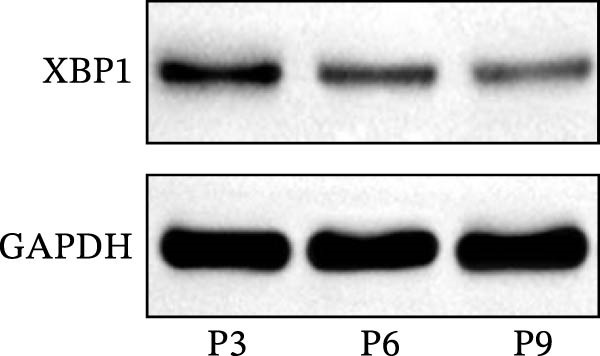
(D)

**Figure:**
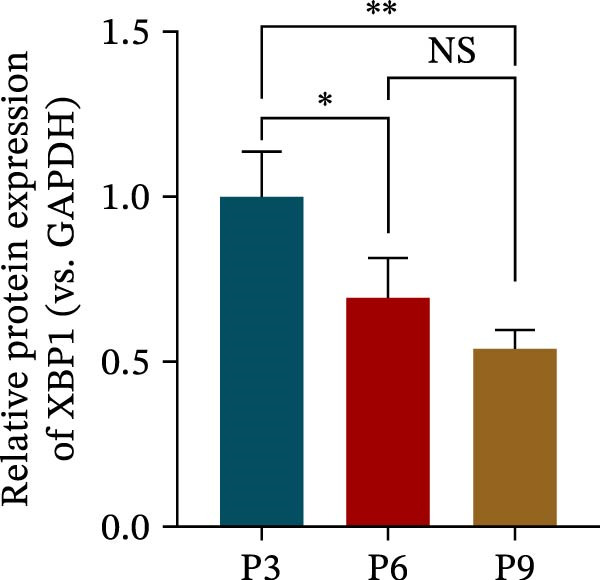
(E)

**Figure:**
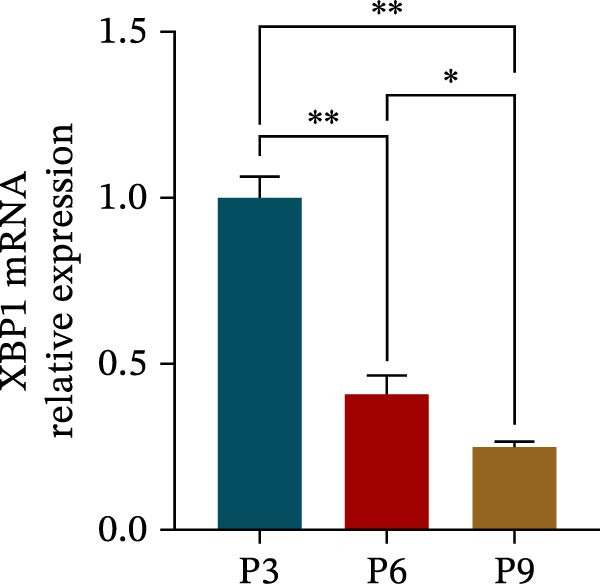
(F)

**Figure:**
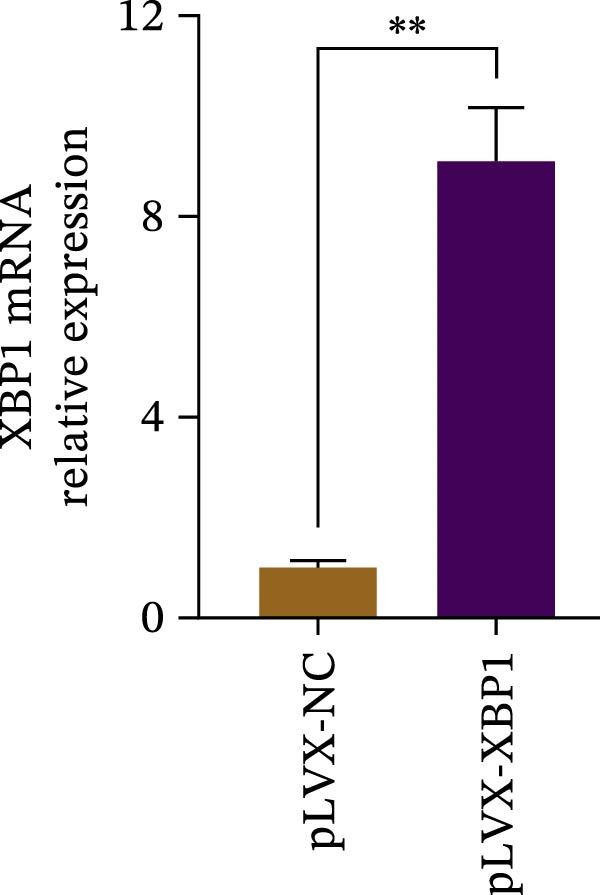
(G)

**Figure:**
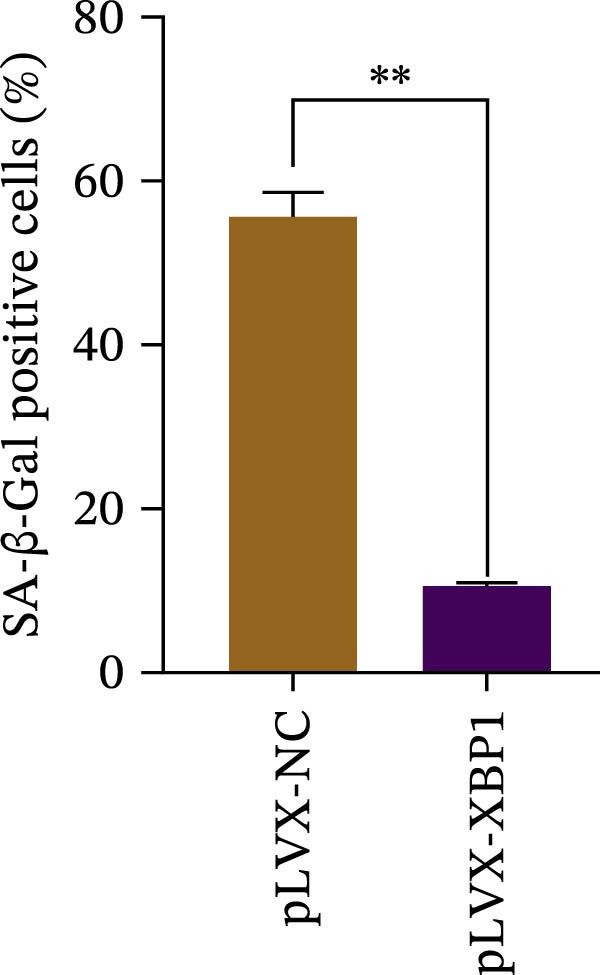
(H)

**Figure:**
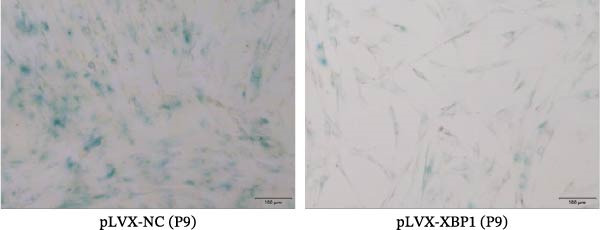
(I)

**Figure:**
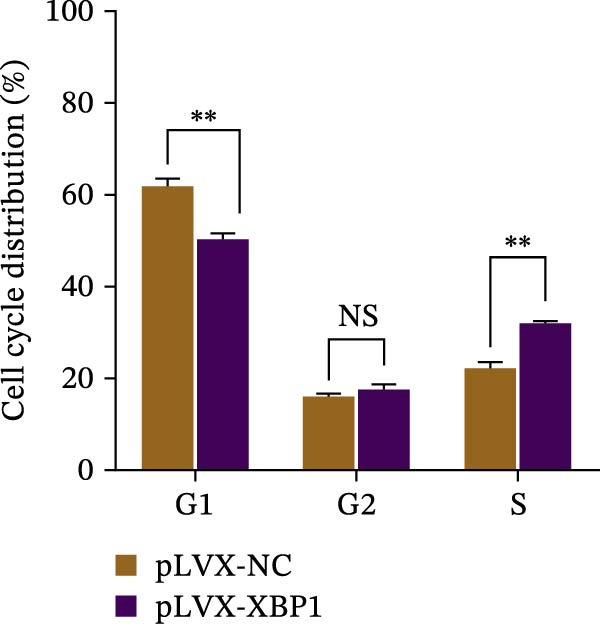
(J)

**Figure:**
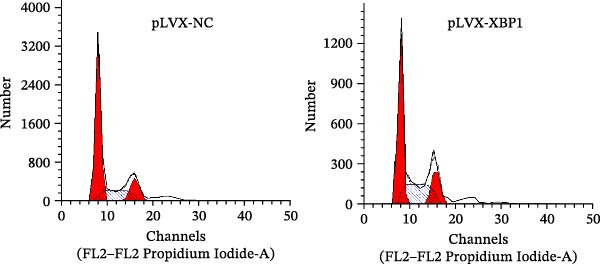
(K)

### 3.4. XBP1 Enhances Osteogenic Differentiation Ability of PDLSCs

To further elucidate the role of XBP1 in the osteogenic differentiation of PDLSCs, we examined the expression of XBP1 in PDLSCs subjected to osteogenic induction. Our results revealed a significant upregulation of both XBP1 protein and mRNA levels following osteogenic differentiation (Figure 4A–C). Moreover, overexpression of XBP1 in PDLSCs led to a significant upregulation of RUNX2 and OCN expression (Figure 4D–F), an increase in ALP activity (Figure 4G,H), and an enhancement of mineralization (Figure 4I,J).

Figure 4XBP1 enhances osteogenic differentiation ability of PDLSCs. (A–C) Western blotiing (A, B) and RT‐PCR (C) of XBP1 in PDLSCs under osteogenic induction. (D–F) Protein expression levels of RUNX2 and OCN detected by western blot. (G, H) PDLSCs were stained with ALP. (I, J) PDLSCs were stained with alizarin red. All experiments were performed in triplicate, and the bars represent the mean ± SD,  ^∗^ *p* < 0.05,  ^∗∗^ *p* < 0.01.

**Figure:**
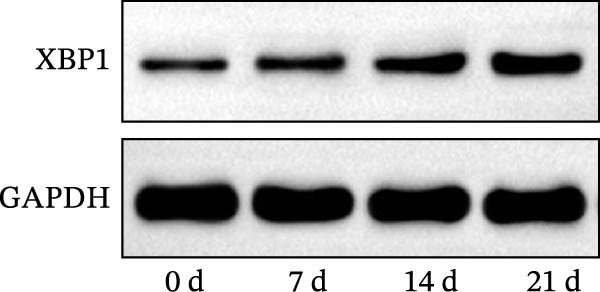
(A)

**Figure:**
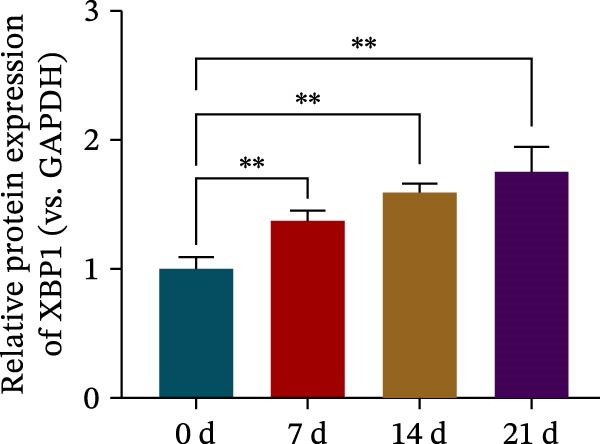
(B)

**Figure:**
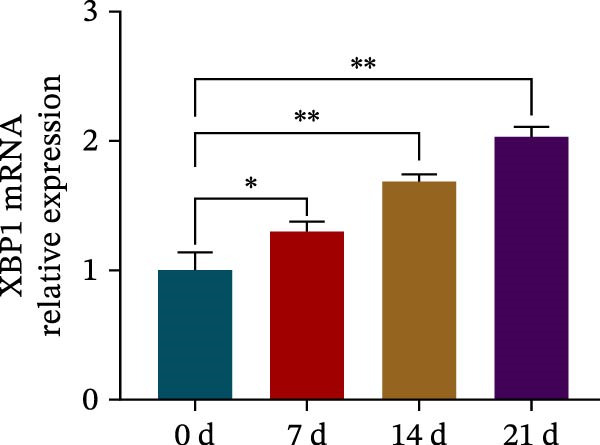
(C)

**Figure:**
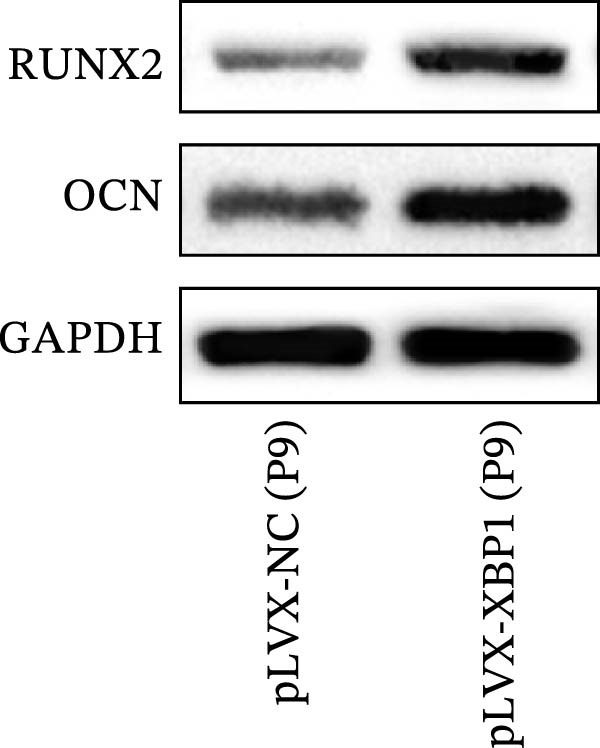
(D)

**Figure:**
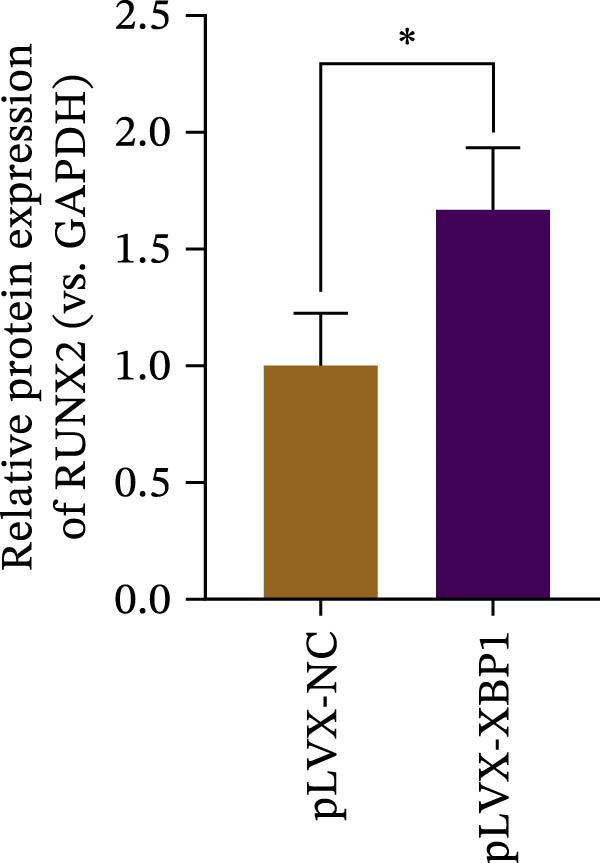
(E)

**Figure:**
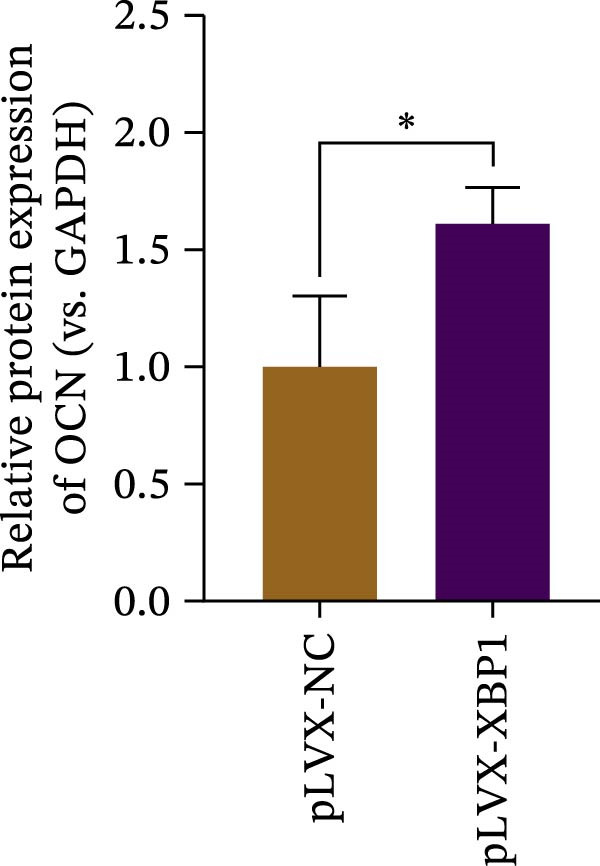
(F)

**Figure:**
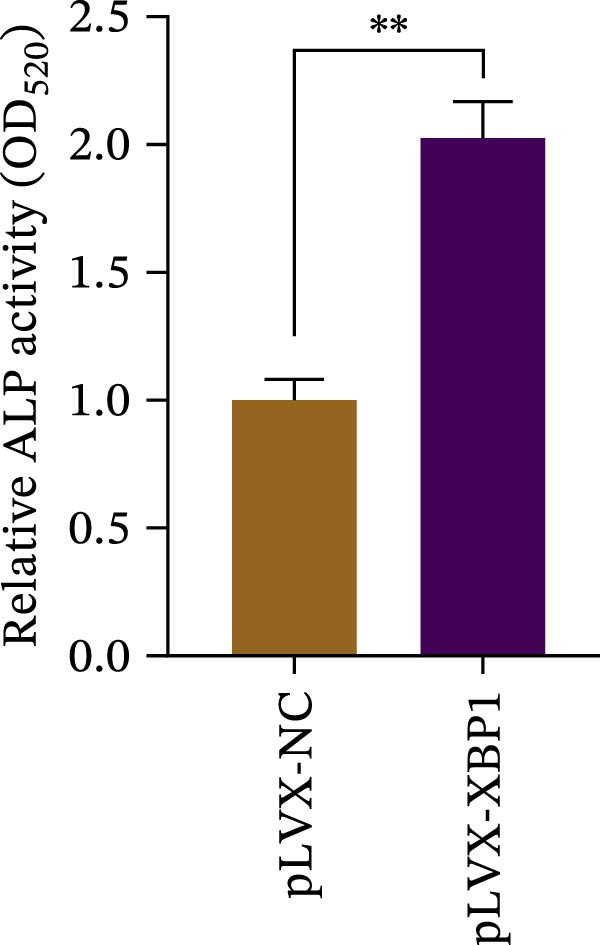
(G)

**Figure:**
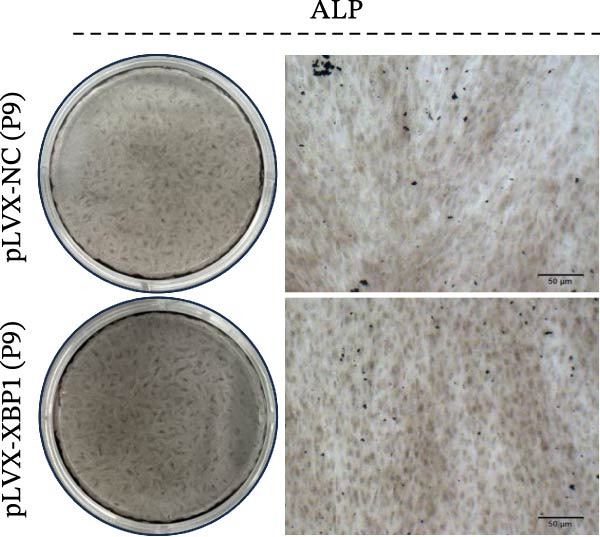
(H)

**Figure:**
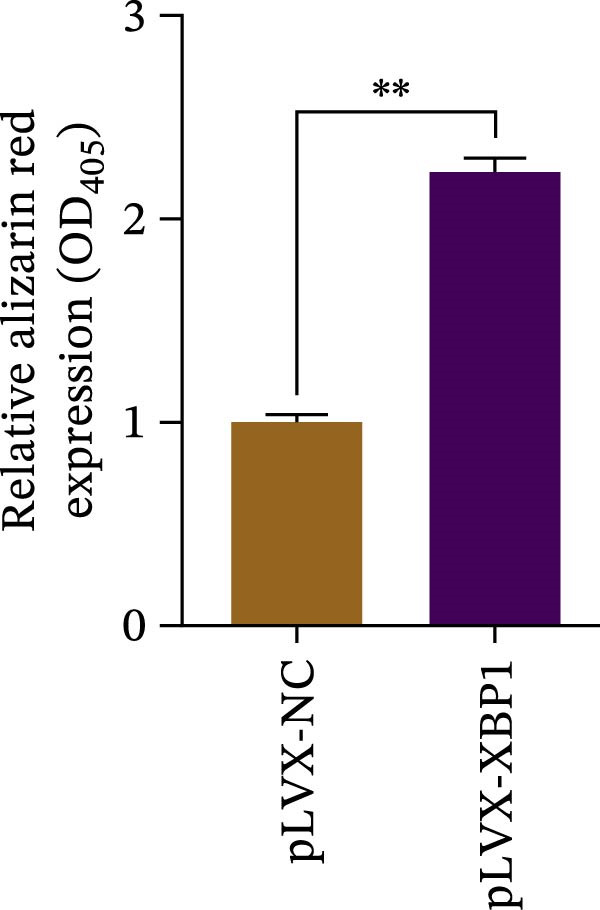
(I)

**Figure:**
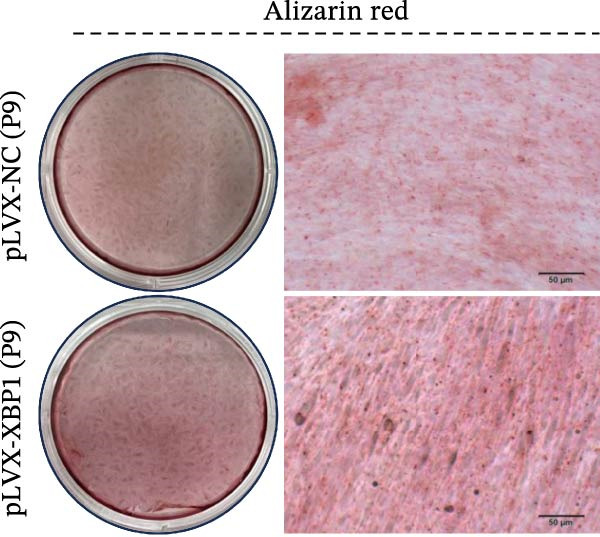
(J)

### 3.5. XBP1 Regulates the Aging‐Related P53 Signaling Pathway

We acquired 8486 target genes of XBP1 from the GTRD database and 1393 osteogenic genes associated with PDLSCs from the GEO database (Figure S1). Through further analysis, we identified 211 target genes that are potentially regulated by XBP1 during the osteogenic differentiation of PDLSCs (Figure 5A). These potential target genes of XBP1 exhibit significant overexpression in the osteogenic differentiation of PDLSCs (Figure 5B and Table S3).

Figure 5XBP1 regulates the aging‐related P53 signaling pathway. (A) Venn diagram of overlapping genes. (B) Heatmap of XBP1 target genes in PDLSCs (top 20). (C) GO enrichment analysis of XBP1 target genes. (D) KEGG pathway enrichment analysis of XBP1 target genes. (E–H) Protein expression levels of P21 and P53 detected by western blot. All experiments were performed in triplicate, and the bars represent the mean ± SD,  ^∗^ *p* < 0.05.

**Figure:**
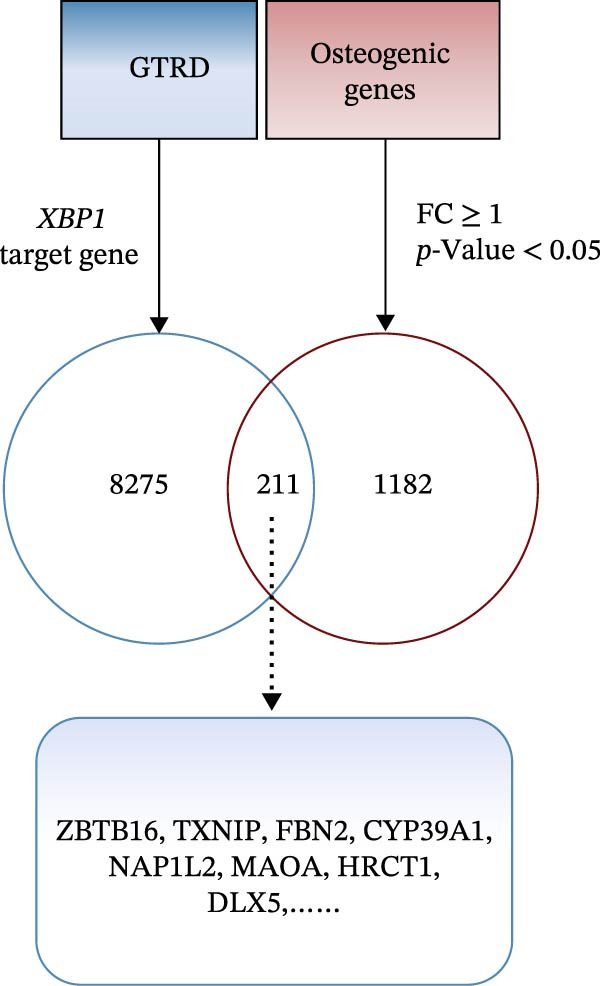
(A)

**Figure:**
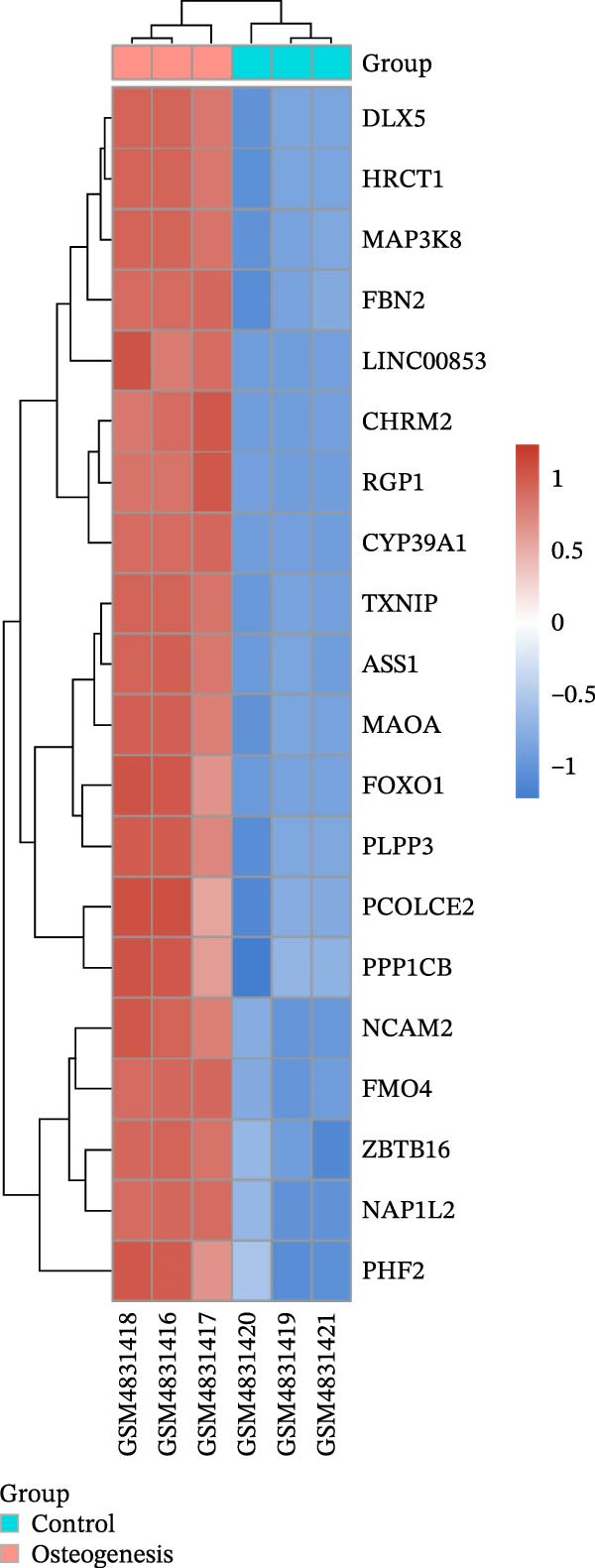
(B)

**Figure:**
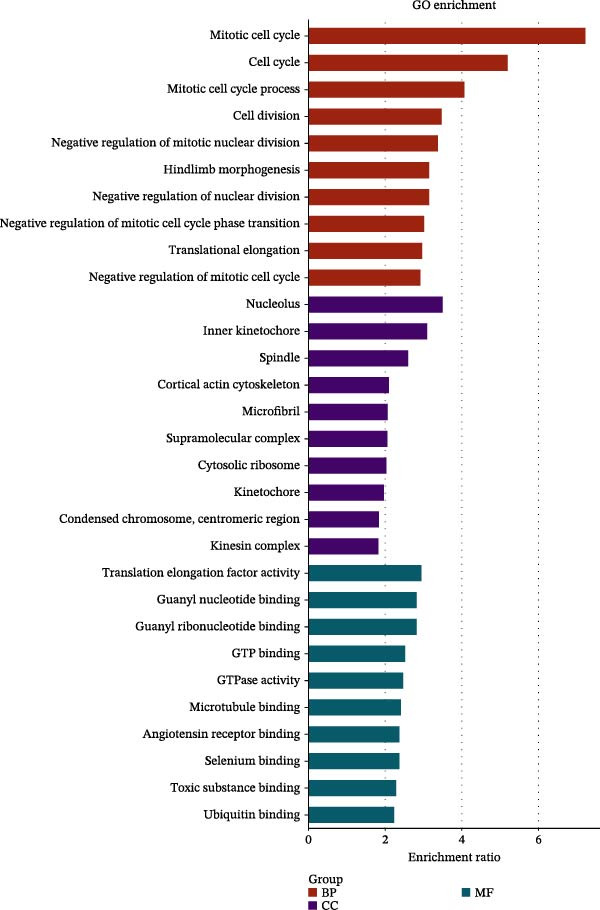
(C)

**Figure:**
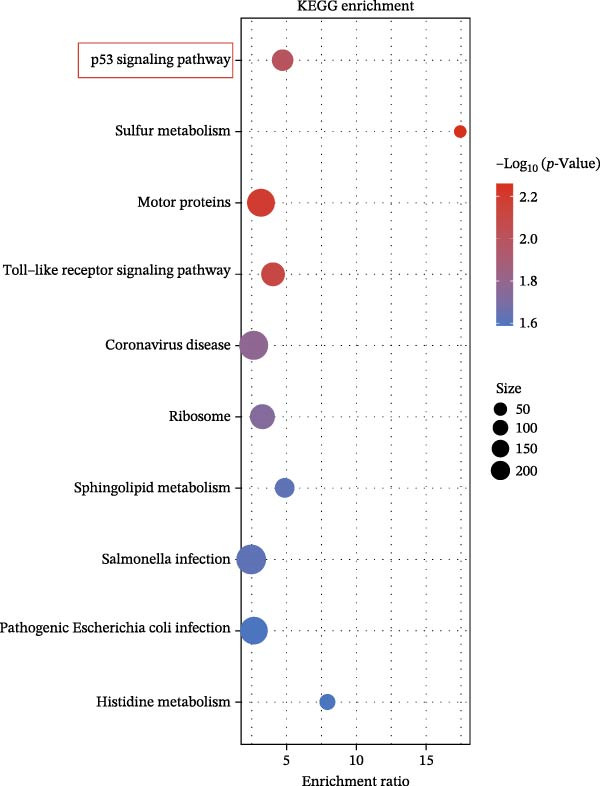
(D)

**Figure:**
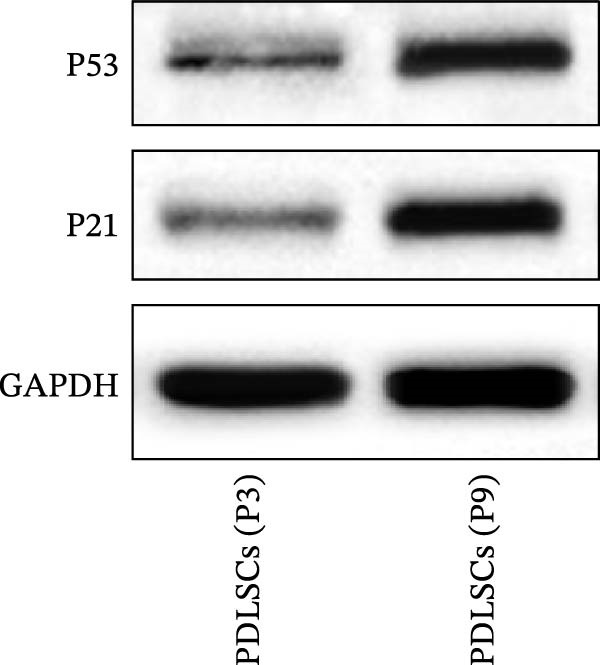
(E)

**Figure:**
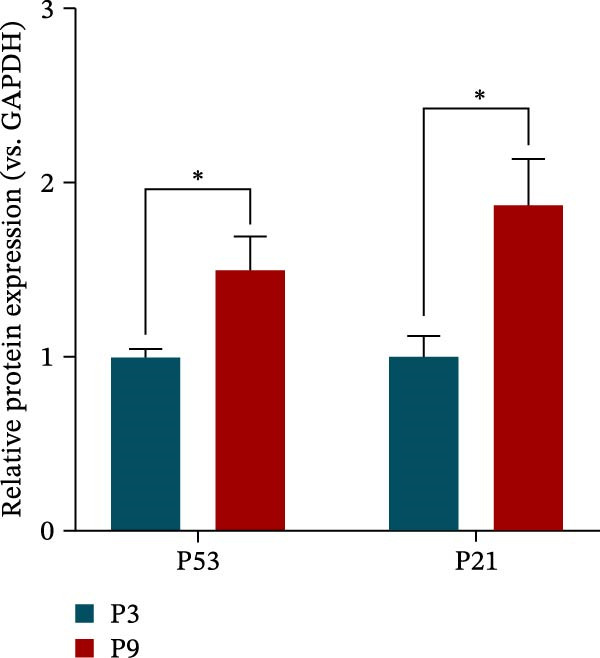
(F)

**Figure:**
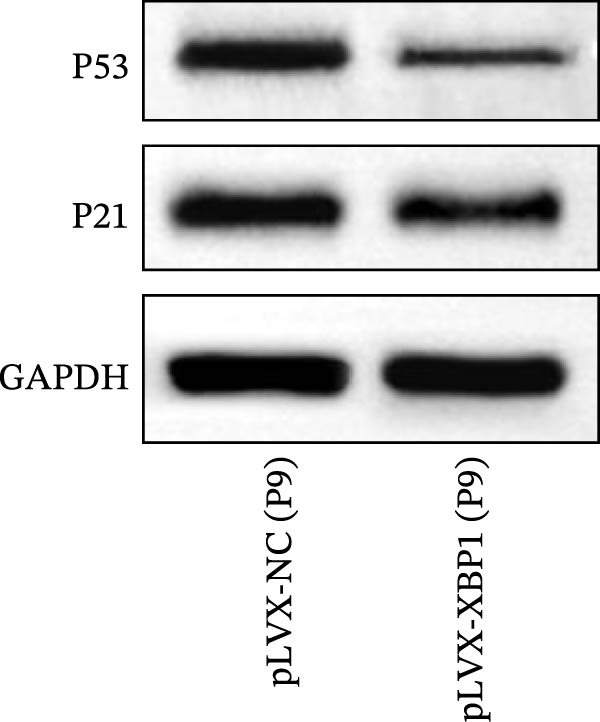
(G)

**Figure:**
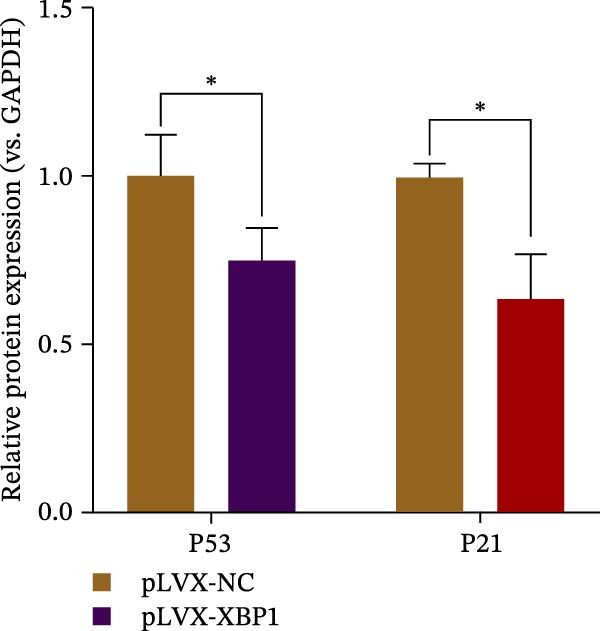
(H)

GO enrichment analysis revealed that these target genes are involved in various PDLSCs cell cycle processes, including mitotic cell cycle, cell cycle, mitotic cell cycle process, negative regulation of mitotic cell cycle, et cetera (Figure 5C). KEGG enrichment analysis further showed that these target genes play roles in multiple PDLSCs signaling pathways, such as P53 signaling pathway and toll‐like receptor signaling pathway, et cetera (Figure 5D). WB analysis confirmed that the expression levels of P53 and its downstream P21 proteins were significantly increased with passage of PDLSCs (Figure 5E,F), while they were significantly decreased after overexpression of XBP1 (Figure 5G,H).

### 3.6. XBP1 Affects Osteogenic Differentiation of PDLSCs Through the P53 Signaling Pathway

ChIP–PCR indicated that the XBP1 antibody could effectively enrich the first site of *P53* promoter region (Figure 6A). Consistently, luciferase reporter assay indicated that XBP1 could bind with the *P53* promoter region, suggesting the XBP1‐mediated inhibition for *P53* transcription (Figure 6B). Third‐passage (P3) PDLSCs were transduced with lentiviral vectors carrying shXBP1, resulting in efficient silencing of the XBP1 gene (Table S4 and Figure S2). WB analysis revealed that silencing of XBP1 led to a significant upregulation of P53 and its downstream target P21 (Figure 6C,D).

Figure 6XBP1 affects osteogenic differentiation of PDLSCs through the P53 signaling pathway. (A) Binding of XBP1 to the P53 promoter was determined using ChIP‐PCR. (B) Luciferase assay was used to determine P53 promoter activity. (C, D) Western blot analysis for the P53 and P21 protein level after the transfection of shXBP1. (E–G) Protein expression levels of RUNX2 and OCN detected by Western blot. (H, I) PDLSCs were stained with ALP. (J, K) PDLSCs were stained with alizarin red. All experiments were performed in triplicate, and the bars represent the mean ± SD,  ^∗^ *p* < 0.05,  ^∗∗^ *p* < 0.01.

**Figure:**
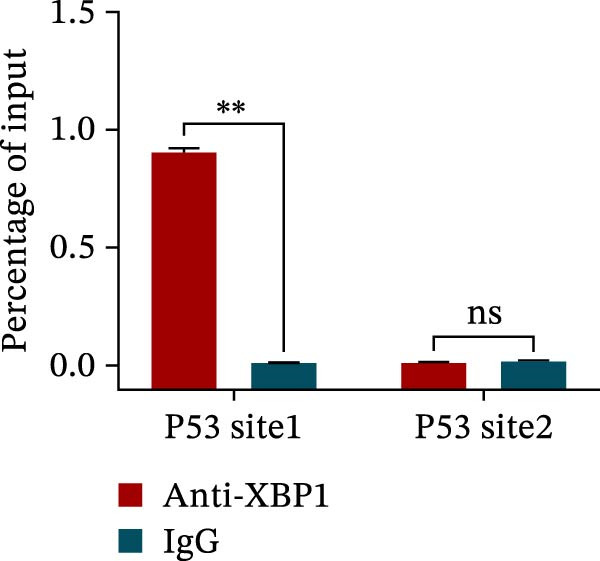
(A)

**Figure:**
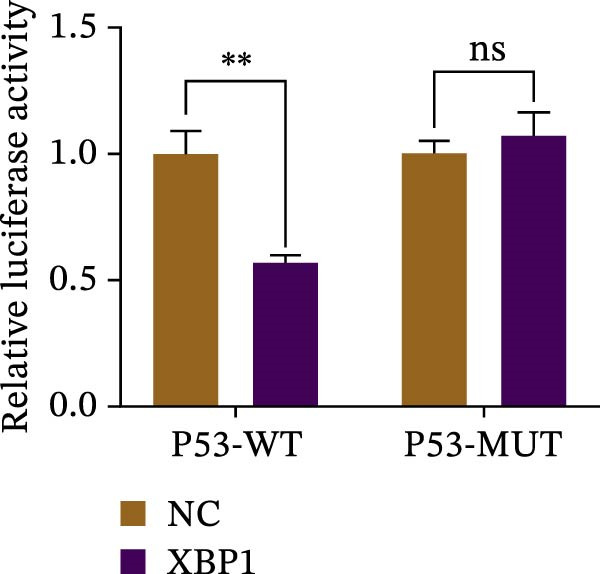
(B)

**Figure:**
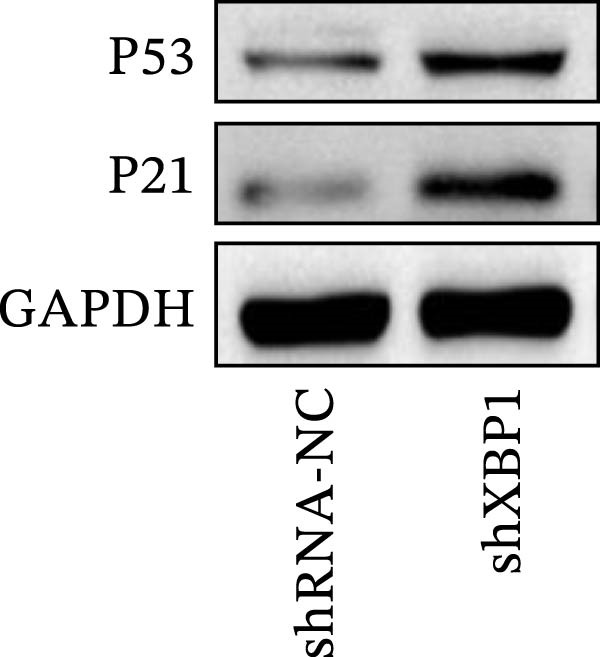
(C)

**Figure:**
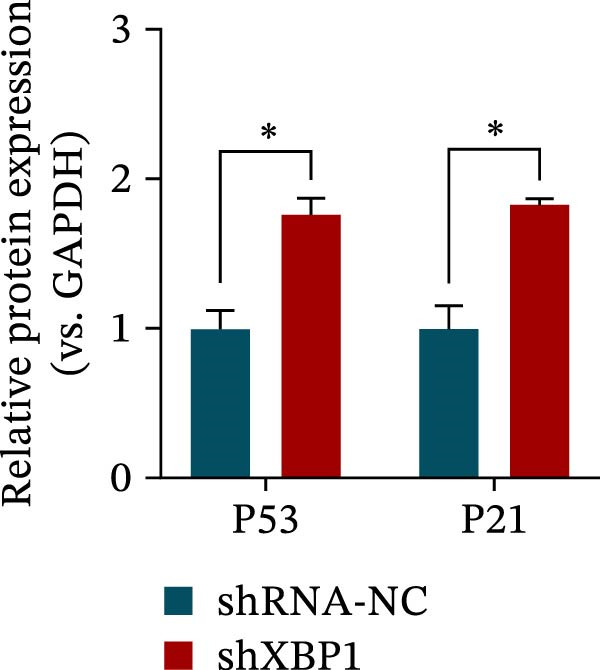
(D)

**Figure:**
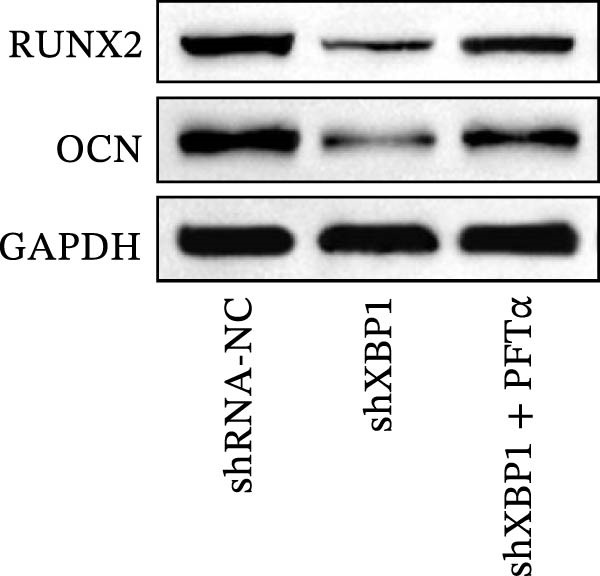
(E)

**Figure:**
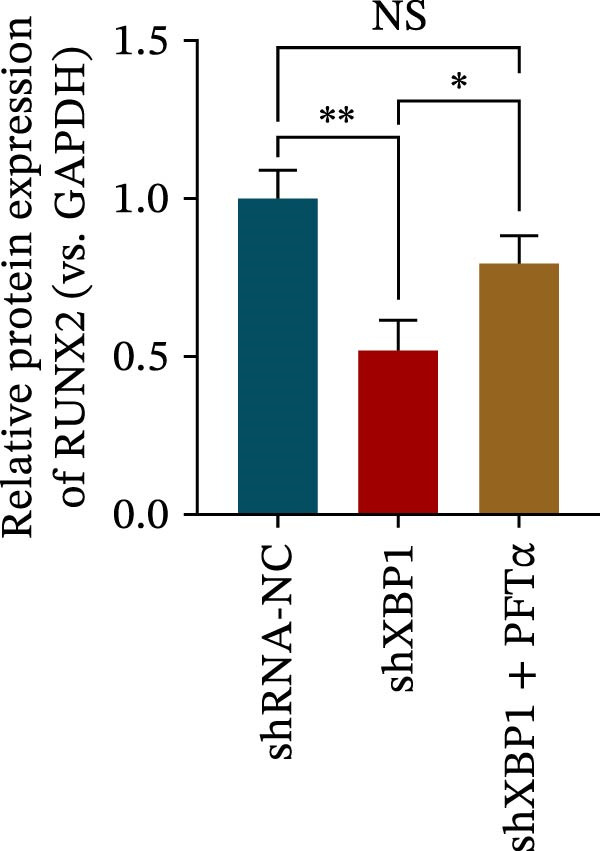
(F)

**Figure:**
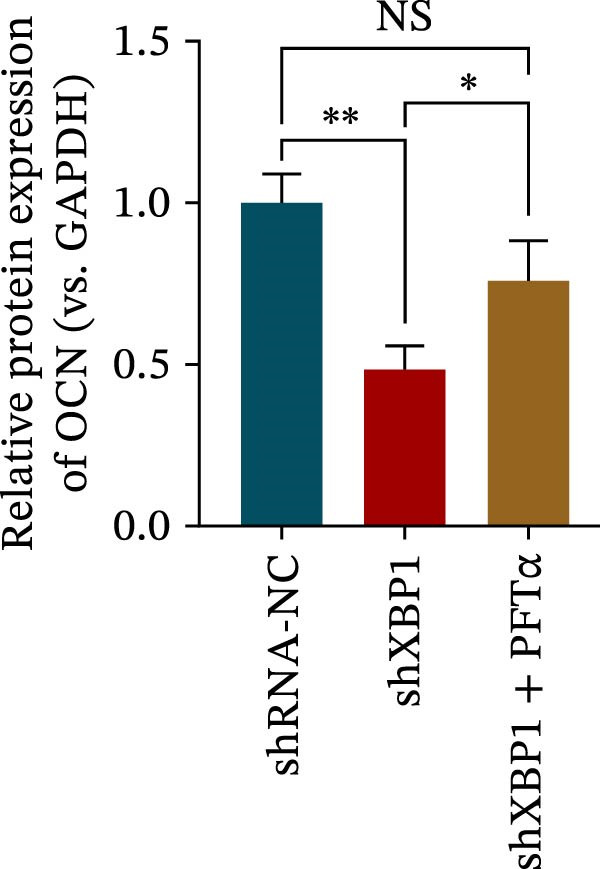
(G)

**Figure:**
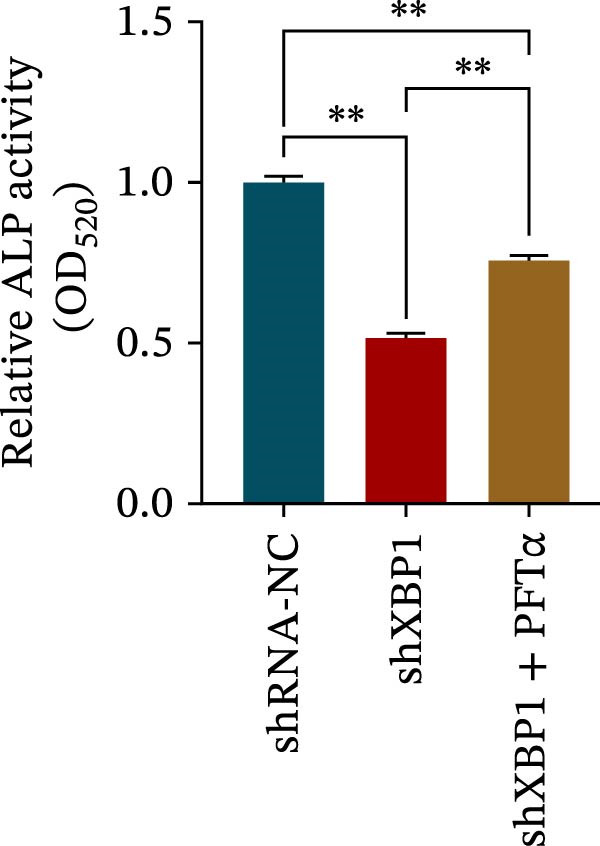
(H)

**Figure:**
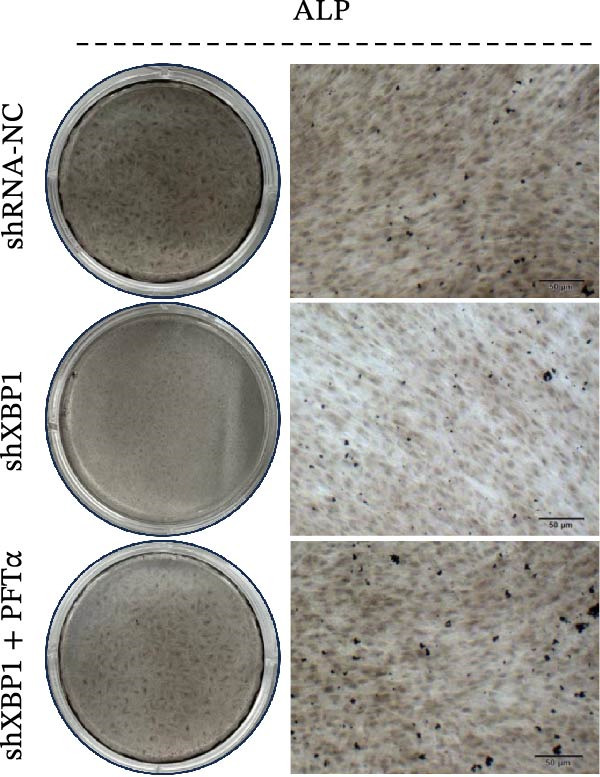
(I)

**Figure:**
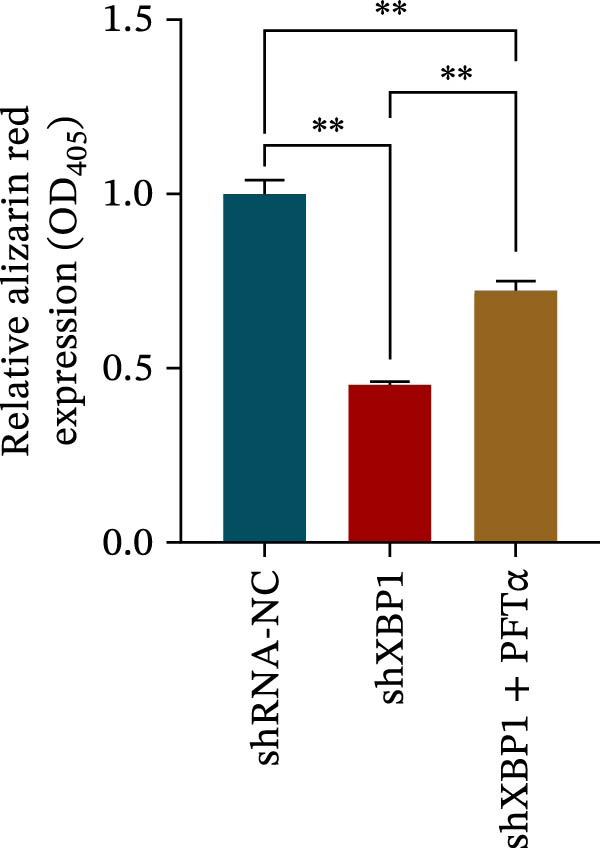
(J)

**Figure:**
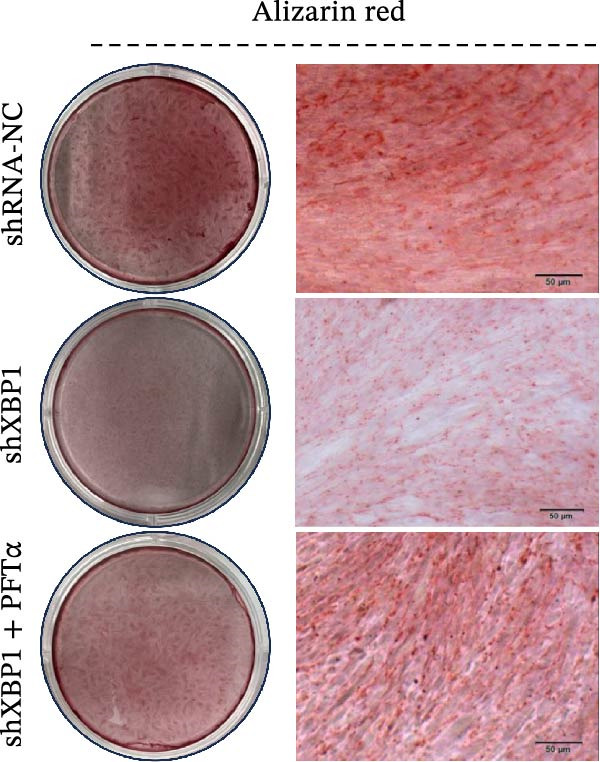
(K)

Functionally, knockdown of XBP1 significantly reduced the expression of RUNX2 and OCN (Figure 6E–G), ALP activity (Figure 6H,I), and mineralization (Figure 6J,K). However, the application of a P53 inhibitor (PFTα, Yeasen Biotech, Shanghai, China) effectively reversed these effects, implying a potential role of P53 in the regulation of XBP1 during osteoblast differentiation.

## 4. Discussion

Cellular senescence is a crucial factor that significantly impacts the vitality and functionality of cells [15]. With advancing age, PDLSCs also experience senescence, a process that impairs their capacity for repair and maintaining tissue homeostasis [8]. Our research identified XBP1, a critical transcription factor, as a key regulator in the aging process of PDLSCs. Notably, we found that the expression of XBP1 declines as PDLSCs age. More intriguingly, upregulating XBP1 expression was shown to partially alleviate this aging process, significantly boosting the osteogenic differentiation potential of these cells.

The fundamental manifestation of cell senescence is characterized by the arrest of the cell cycle, leading to a reduction in mitotic activity, a decline in cell proliferation, and a dampened response to apoptotic signals [16]. Concurrently, as cells age, the expression levels of aging markers progressively increase, with β‐galactosidase being one of the most widely employed indicators of aging [17]. Our study reveals a significant upsurge in the activity of this aging marker, β‐galactosidase, in E‐PDLSCs when compared with Y‐PDLSCs. Moreover, in contrast to Y‐PDLSCs, E‐PDLSCs exhibit an increment in the G1 phase and a decrement in the S phase. Our data suggest that E‐PDLSCs from older subjects exhibit aging cell characteristics, highlighting their potential as a model to study aging mechanisms.

Transcription factors are pivotal in regulating gene expression and influencing multiple drivers of aging, thereby playing a crucial role in determining the lifespan of cells [18]. XBP1, in particular, has emerged as a key player in the aging process of various cells and organs, as evidenced by numerous studies involving macrophages [11], intestinal stem cells [10], the hippocampus [12], and the cardio‐renal system [13]. To further explore the role of XBP1 in the aging dynamics of PDLSCs, we conducted a series of experiments. Our findings revealed a decline in XBP1 expression levels in aging PDLSCs. Moreover, when XBP1 was overexpressed in PDLSCs, it significantly alleviated the aging process, as demonstrated by a reduction in β‐galactosidase expression, a decrease in the S1 phase, and an increase in the S phase. These results suggest that XBP1 may hold promise as a potential target for mitigating age‐related cellular decline in PDLSCs.

With the increase of age, the expression of osteogenic marker protein and the activity of ALP in periodontal ligament cells decreased, which ultimately reduced the repair ability of periodontal tissue [19]. PDLSCs from elderly donors not only demonstrated decreased proliferation, migration, and differentiation ability in vitro, but also showed significantly decreased regeneration ability in vivo compared to those from younger donors [8]. Our study further confirms these findings, indicating that aging PDLSCs indeed exhibit impaired osteogenic differentiation capabilities. Given the pivotal role of XBP1 in regulating the aging process of PDLSCs, we delved deeper into its influence on their osteogenic differentiation. Our findings revealed that overexpressing XBP1 in PDLSCs significantly enhanced their osteogenic differentiation potential. Future studies should explore the molecular mechanisms by which XBP1 exerts its effects on the osteogenic differentiation of PDLSCs.

To gain a deeper insight into whether the regulatory effect of XBP1 on the osteogenic capacity of PDLSCs is associated with the aging process, we further screened the potential target genes of XBP1 in PDLSCs. Our findings revealed that these genes are significantly enriched in various cell cycle processes. Importantly, cellular senescence is precisely characterized as an irreversible cell cycle arrest phenomenon [20]. Cell growth arrest often entails the intricate interplay of multiple signaling pathways, with the P53/P21 pathway emerging as a pivotal player in initiating cellular senescence, particularly during the early stages [21]. Intriguingly, our findings indicate that the potential target gene of XBP1 in PDLSCs is notably enriched within the P53/P21 pathway. This suggests that XBP1’s modulation of PDLSCs’ osteogenic capacity is intricately linked with the aging process and may be executed via the P53/P21 pathway. Subsequent experiments reinforced this hypothesis by demonstrating that suppressing the P53/P21 pathway could notably enhance the diminished osteogenic potential of PDLSCs resulting from reduced XBP1 expression.

We must be cognizant of several limitations inherent in this study. Periodontitis is a chronic inflammatory disease driven by the complex interplay of multiple cell types. XBP1, a transcription factor expressed in plasma cells, multiple myeloma cells, and bone marrow stromal cells, plays a dual role in bone metabolism by regulating both osteoblast and osteoclast differentiation [22]. In macrophages, XBP1 promotes the production of inflammatory cytokines and drives polarization toward the pro‐inflammatory M1 phenotype [23]. In CD4^+^ T cells, it facilitates T helper (Th) cell polarization, leading to the secretion of cytokine [24]. Furthermore, in CD8^+^ T cells, XBP1 regulates the expression of cholesterol‐induced inhibitory receptors, contributing to T cell exhaustion [25]. However, the specific roles of XBP1 in these cells involved in the pathogenesis of periodontitis remain to be fully elucidated. Second, our research was confined to the cellular level, and the impact of XBP1 on aging patients with periodontitis remains obscure. Third, despite the significant enrichment of the XBP1 target gene in the P53/P21 pathway, other pathways are likely involved in the regulation of XBP1, warranting further exploration through additional studies. Future studies should aim to address the aforementioned limitations by investigating the effects of XBP1 on periodontitis in animal models and clinical settings.

## 5. Conclusion

In conclusion, our study demonstrates that XBP1 plays a pivotal role in regulating the osteogenic differentiation potential of PDLSCs during aging. These findings provide new insights into the mechanisms underlying the age‐related decline in periodontal tissue repair and suggest that XBP1 may be a promising target for developing therapeutic strategies to enhance periodontal tissue regeneration in aging individuals.

## Funding

This study was supported by the Tianjin Health Research Project (Grant TJWJ2023MS034), the Natural Science Foundation of Tianjin, China (Grant 23JCYBJC01130), and the Science and Technology Foundation of Tianjin Stomatological Hospital (Grant 2023KLQN05).

## Conflicts of Interest

The authors declare no conflicts of interest.

## Supporting Information

Additional supporting information can be found online in the Supporting Information section.

## Supporting information


**Supporting Information** Table S1. Primer sequences for RT‐qPCR. Table S2. The potential target genes of XBP1 in PDLSCs. Table S3. Three shRNAs targeting human XBP1 were designed. Figure S1. Osteogenic genes were identified and screened from the GEO database (GSE159507). Figure S2. RT‐PCR analysis of XBP1 in PDLSCs infected with lentivirus.

## Data Availability

The datasets generated and/or analyzed during the current study are available from the corresponding author upon reasonable request.
