# Supplementary material for: X‐Box Binding Protein 1 Regulates Osteogenesis of Periodontal Ligament Cells During Aging by Modulating P53 Signaling Pathway
Source: Stem Cells Int. 2026 Apr 10;2026:2846096. doi: 10.1155/sci/2846096 (PMC13067754; doi:10.1155/sci/2846096)
Supplement: Supplementary file 1 — Supporting Information Table S1. Primer sequences for RT‐qPCR. Table S2. The potential target genes of XBP1 in PDLSCs. Table S3. Three shRNAs targeting human XBP1 were designed. Figure S1. Osteogenic genes were identified and screened from the GEO database (GSE159507). Figure S2. RT‐PCR analysis of XBP1 in PDLSCs infected with lentivirus. [file SCI-2026-2846096-s001.docx]

**Supplementary Materials**

**SUPPLEMENTARY TABLE 1** Primer sequences for RT-qPCR.

| Primer | Sequence |
| --- | --- |
| XBP1 | F: CCCTCCAGAACATCTCCCCAT  R: ACATGACTGGGTCCAAGTTGT |
| GAPDH | F: GCACCGTCAAGGCTGAGAAC  R: TGGTGAAGACGCCAGTGGA |

XBP1, X-box binding protein 1; F, forward; R, reverse.

**SUPPLEMENTARY TABLE 2** Primers used for ChIP PCR.

| Primer | Sequence |
| --- | --- |
| P53 site1 | F: GCGGACGCCAATTCTTTTGA  R: CTAGGGCTTGATGGGAACGG |
| P53 site2 | F: TCTCCCCAGACTCCACACTC  R: AGTTGGGGTCTGGAAAAGCC |

F, forward; R, reverse.

**SUPPLEMENTARY TABLE 3** The potential target genes of XBP1 in PDLSCs.

| Gene symbol | P.Value | logFC | Gene symbol | P.Value | logFC | Gene symbol | P.Value | logFC | Gene symbol | P.Value | logFC |
| --- | --- | --- | --- | --- | --- | --- | --- | --- | --- | --- | --- |
| ZBTB16 | 9.65E-06 | 9.15 | SLC16A14 | 0.00208 | 1.87 | TAF9B | 0.000252 | 1.33 | CNTROB | 2.98E-05 | 1.15 |
| TXNIP | 4.08E-08 | 7.96 | FHIT | 2.21E-06 | 1.86 | MEX3B | 0.00956 | 1.32 | TMEM232 | 0.000438 | 1.15 |
| FBN2 | 8.01E-07 | 5.77 | DDIT4 | 1.04E-05 | 1.85 | ANKEF1 | 4.41E-06 | 1.31 | TRA2A | 0.0169 | 1.15 |
| CYP39A1 | 1.01E-09 | 5.5 | SLC25A27 | 1.97E-06 | 1.82 | ANLN | 0.000897 | 1.31 | EEF1A1 | 2.12E-05 | 1.13 |
| NAP1L2 | 6.88E-06 | 4.94 | HSPB1 | 6.43E-06 | 1.79 | TUBB3 | 1.07E-06 | 1.3 | DZIP3 | 1.82E-06 | 1.12 |
| MAOA | 1.3E-06 | 4.53 | MIR940 | 6.42E-05 | 1.78 | EIF5A2 | 5.45E-06 | 1.3 | CEP68 | 1.78E-05 | 1.12 |
| HRCT1 | 1.02E-06 | 4.32 | PKIB | 0.00101 | 1.77 | ME1 | 7.96E-05 | 1.3 | RAB40C | 2.01E-05 | 1.12 |
| DLX5 | 4.92E-07 | 3.9 | TWIST1 | 0.000336 | 1.76 | GDF5 | 2.09E-06 | 1.29 | DDB2 | 0.000153 | 1.12 |
| CHRM2 | 2.93E-07 | 3.76 | GAS2 | 0.0198 | 1.76 | MMP13 | 3.58E-05 | 1.29 | RAB31 | 0.000522 | 1.12 |
| LINC00853 | 4.55E-07 | 3.62 | SUOX | 0.000445 | 1.73 | TK1 | 0.000209 | 1.29 | SPON2 | 0.000634 | 1.12 |
| ASS1 | 2.64E-07 | 3.25 | SPC24 | 4.23E-05 | 1.72 | RAB40B | 0.000964 | 1.29 | SLC19A1 | 0.0013 | 1.12 |
| PLPP3 | 1.12E-05 | 2.99 | REV3L | 0.00045 | 1.71 | VPS37B | 0.000295 | 1.28 | CENPI | 1.59E-06 | 1.11 |
| RGP1 | 3.4E-07 | 2.97 | SRRM3 | 2.31E-06 | 1.7 | MED19 | 0.000756 | 1.28 | NEIL3 | 2.26E-06 | 1.11 |
| PPP1CB | 0.000189 | 2.86 | CNTNAP2 | 0.000512 | 1.69 | CD160 | 0.00809 | 1.28 | TENM3 | 0.000189 | 1.11 |
| MAP3K8 | 5.09E-07 | 2.84 | METTL7A | 0.0419 | 1.68 | TANC1 | 0.000105 | 1.27 | SGMS2 | 0.000608 | 1.11 |
| FMO4 | 3.77E-07 | 2.83 | MDM2 | 4.53E-05 | 1.67 | EEF2K | 0.000375 | 1.27 | DBP | 4.58E-05 | 1.1 |
| FOXO1 | 1.23E-05 | 2.83 | GIPC2 | 0.00245 | 1.67 | BHLHB9 | 0.00439 | 1.27 | ATG10 | 0.000304 | 1.1 |
| PHF2 | 0.000133 | 2.78 | MNS1 | 8.62E-07 | 1.66 | TDO2 | 0.00621 | 1.26 | ZDHHC9 | 0.000014 | 1.09 |
| PCOLCE2 | 0.000121 | 2.73 | ADAMTS1 | 4.44E-05 | 1.66 | EVA1C | 0.00018 | 1.25 | FAHD2A | 3.74E-05 | 1.09 |
| NCAM2 | 3.9E-06 | 2.69 | INTS1 | 0.00194 | 1.66 | ARL4A | 1.16E-06 | 1.24 | YOD1 | 6.36E-05 | 1.09 |
| TCEA3 | 1.12E-07 | 2.6 | HSD17B6 | 6.18E-07 | 1.65 | KDELC1 | 3.44E-06 | 1.24 | DCAKD | 0.000154 | 1.09 |
| LAD1 | 0.000324 | 2.6 | GSR | 1.34E-05 | 1.65 | MTERF2 | 3.23E-05 | 1.24 | ZNF836 | 0.00022 | 1.09 |
| ZNF391 | 2.06E-06 | 2.49 | MMD | 5.91E-05 | 1.65 | MIPOL1 | 0.000158 | 1.24 | RPS2 | 0.000266 | 1.09 |
| NOD1 | 0.000088 | 2.44 | B3GALT4 | 3.02E-06 | 1.63 | CDCA8 | 0.000286 | 1.24 | TPGS2 | 1.63E-05 | 1.08 |
| AP1S2 | 0.00249 | 2.37 | C1orf21 | 2.58E-05 | 1.61 | DIO2 | 0.000557 | 1.24 | EPHX2 | 2.26E-05 | 1.08 |
| RAB25 | 3.43E-05 | 2.35 | CDCA3 | 6.78E-07 | 1.6 | IFNAR2 | 0.000605 | 1.24 | CENPH | 2.89E-05 | 1.08 |
| RGS22 | 7.19E-07 | 2.27 | LRRC31 | 0.00117 | 1.58 | AC098820.2 | 0.00127 | 1.24 | RNF141 | 5.41E-05 | 1.08 |
| CUTC | 3.4E-08 | 2.25 | GSTA4 | 3.4E-07 | 1.57 | KIFC1 | 3.46E-06 | 1.23 | CARNMT1 | 0.0233 | 1.08 |
| PITX1 | 0.000484 | 2.25 | SLC25A23 | 8.59E-07 | 1.57 | CDC42EP4 | 0.000216 | 1.23 | RPL13 | 3.71E-06 | 1.07 |
| USP13 | 1.64E-06 | 2.23 | PRMT2 | 3.24E-06 | 1.56 | RPL12 | 2.97E-06 | 1.22 | RPA3 | 3.45E-05 | 1.07 |
| DEPTOR | 9.88E-08 | 2.21 | LTF | 8.72E-06 | 1.54 | SDHAF3 | 3.85E-06 | 1.22 | CEP350 | 0.00217 | 1.07 |
| TUBB2A | 1.34E-06 | 2.21 | RDX | 0.000574 | 1.54 | MAP2K6 | 4.67E-06 | 1.22 | BCKDHB | 4.32E-05 | 1.06 |
| DNAJB4 | 2.23E-06 | 2.2 | SIDT2 | 1.94E-05 | 1.53 | ZFP2 | 4.46E-05 | 1.22 | TBL1XR1 | 8.76E-05 | 1.06 |
| RNF44 | 9.94E-05 | 2.2 | RPS10-NUDT3 | 1.48E-06 | 1.52 | SYCE2 | 0.00317 | 1.22 | C4orf33 | 3.89E-05 | 1.05 |
| GCNT1 | 9.12E-07 | 2.19 | EEF2 | 3.33E-05 | 1.48 | MTURN | 2.86E-06 | 1.21 | MAD2L2 | 6.47E-05 | 1.05 |
| CMBL | 8.39E-05 | 2.18 | LIPA | 0.0134 | 1.48 | CCNB2 | 9.11E-06 | 1.2 | LYRM7 | 0.000398 | 1.05 |
| APOL2 | 0.0249 | 2.18 | MORN5 | 0.0181 | 1.47 | YWHAZ | 0.00726 | 1.2 | RACGAP1 | 0.00164 | 1.05 |
| SELENBP1 | 9.1E-06 | 2.17 | TSHZ1 | 1.48E-06 | 1.46 | PRSS12 | 4.67E-05 | 1.19 | MYO1E | 0.00233 | 1.05 |
| UNC13B | 2.44E-05 | 2.14 | LGI2 | 1.85E-05 | 1.46 | NEK3 | 0.000228 | 1.19 | CXXC5 | 3.08E-06 | 1.04 |
| SIPA1L3 | 1.33E-07 | 2.13 | CHSY3 | 9.46E-06 | 1.41 | SMYD4 | 0.00171 | 1.19 | CENPP | 3.97E-06 | 1.04 |
| KIF20A | 3.61E-06 | 2.13 | RFC4 | 0.000314 | 1.41 | RPL10L | 3.83E-05 | 1.18 | ACER3 | 0.000958 | 1.04 |
| TLR5 | 2.14E-05 | 2.13 | PCDHGC3 | 0.0116 | 1.41 | RBBP9 | 7.58E-05 | 1.18 | HS1BP3 | 0.008 | 1.04 |
| PIR | 4.28E-06 | 2.12 | TYK2 | 0.0235 | 1.4 | RETSAT | 0.00287 | 1.18 | SPC25 | 3.62E-06 | 1.03 |
| RASD1 | 0.00137 | 2.11 | MACROD1 | 2.07E-05 | 1.39 | KIF15 | 0.000573 | 1.17 | RPL23AP7 | 1.19E-05 | 1.03 |
| ANKFN1 | 0.000187 | 2 | HERPUD2 | 0.00151 | 1.39 | SOCS3 | 0.00321 | 1.17 | GTSE1 | 0.0248 | 1.03 |
| HNRNPH1 | 0.00151 | 2 | TUBA1C | 1.65E-06 | 1.37 | NAP1L3 | 0.00357 | 1.17 | VPS51 | 5.72E-06 | 1.02 |
| CRYAB | 2.61E-05 | 1.98 | USP53 | 0.0107 | 1.37 | CC2D1A | 0.0473 | 1.17 | PIPOX | 0.000124 | 1.02 |
| C1QBP | 1.87E-07 | 1.97 | TOM1L1 | 7.75E-07 | 1.35 | RNH1 | 3.9E-06 | 1.16 | ELOVL5 | 0.000231 | 1.02 |
| FBXO36 | 1.24E-06 | 1.94 | DRAM2 | 1.38E-05 | 1.35 | LGMN | 0.000105 | 1.16 | PBK | 0.000012 | 1.01 |
| IL18 | 9.29E-05 | 1.93 | VRTN | 0.000168 | 1.35 | UBR3 | 0.000173 | 1.16 | EFEMP2 | 1.29E-05 | 1.01 |
| KLHL17 | 3.83E-06 | 1.9 | TRPC1 | 0.000186 | 1.35 | PPP5D1 | 0.00134 | 1.16 | CTDSP1 | 0.000414 | 1.01 |
| ZNF646 | 0.00234 | 1.89 | EPHB3 | 5.3E-06 | 1.34 | STOML1 | 0.00158 | 1.16 | ERGIC1 | 0.000464 | 1.01 |
| TSPAN8 | 4.04E-05 | 1.87 | FBXO32 | 9.7E-06 | 1.33 | BLMH | 2.71E-06 | 1.15 |  |  |  |

FC, fold change.

**SUPPLEMENTARY TABLE 4** Three shRNAs targeting human XBP1 were designed.

| Primer | Primer SeqTence |
| --- | --- |
| shXBP1-1 | F: GATCCGCGGTATTGACTCTTCAGATTCTCGAGAATCTGAAGAGTCAATACCGCTTTTTG  R: AATTCAAAAAGCGGTATTGACTCTTCAGATTCTCGAGAATCTGAAGAGTCAATACCGCG |
| shXBP1-2 | F: GATCCGAACAGCAAGTGGTAGATTTACTCGAGTAAATCTACCACTTGCTGTTCTTTTTG  R: AATTCAAAAAGAACAGCAAGTGGTAGATTTACTCGAGTAAATCTACCACTTGCTGTTCG |
| shXBP1-3 | F: GATCCGACCCAGTCATGTTCTTCAAACTCGAGTTTGAAGAACATGACTGGGTCTTTTTG  R: AATTCAAAAAGACCCAGTCATGTTCTTCAAACTCGAGTTTGAAGAACATGACTGGGTCG |

XBP1, X-box binding protein 1; F, forward; R, reverse.


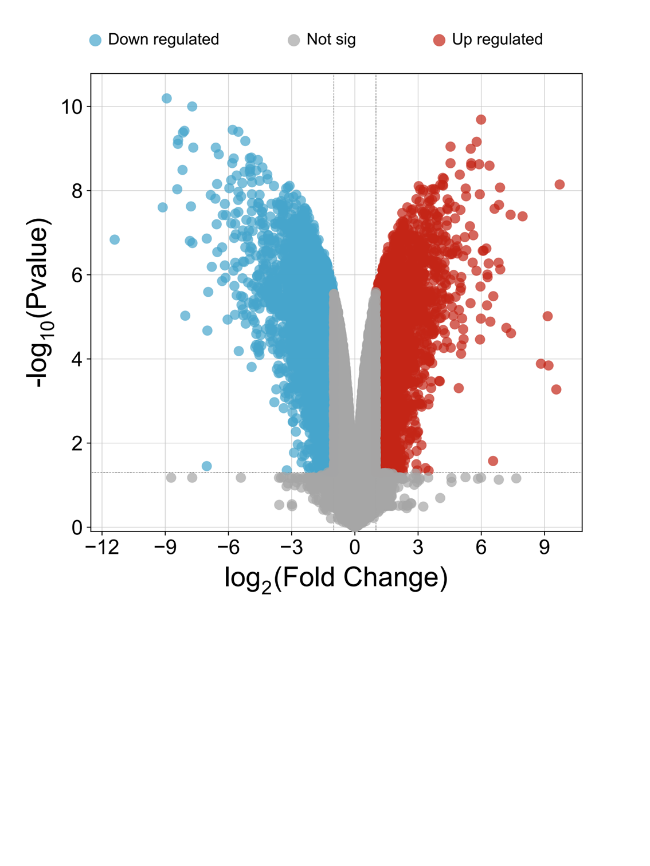


**SUPPLEMENTARY FIGURE 1** Osteogenic genes were identified and screened from the GEO database (GSE159507). |log2 fold change| > log2 1.5 and P-value < 0.05.


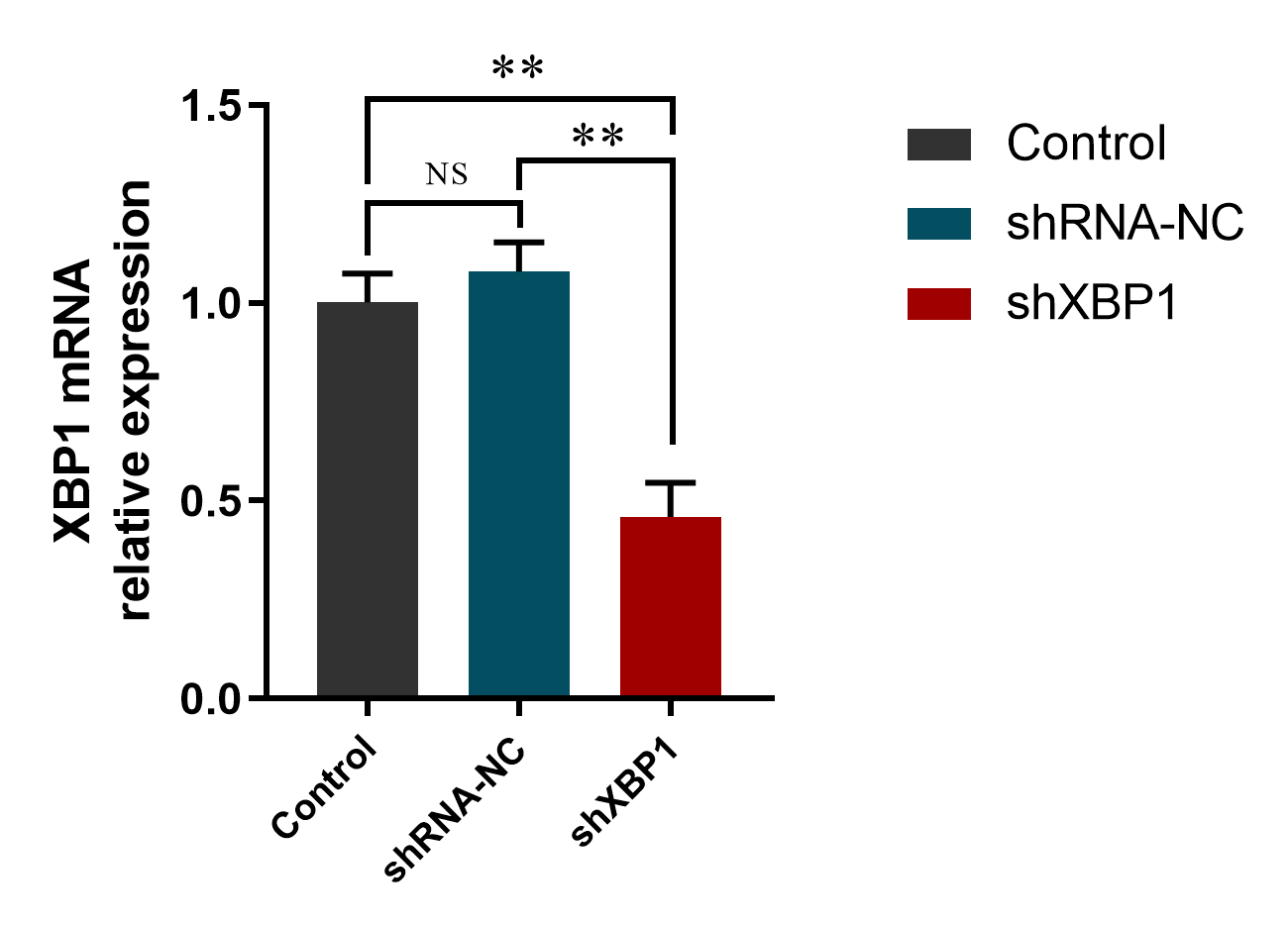


**SUPPLEMENTARY FIGURE 2** RT-PCR analysis of XBP1 in PDLSCs infected with lentivirus
